# CNVassoc: Association analysis of CNV data using R

**DOI:** 10.1186/1755-8794-4-47

**Published:** 2011-05-24

**Authors:** Isaac Subirana, Ramon Diaz-Uriarte, Gavin Lucas, Juan R Gonzalez

**Affiliations:** 1 CIBER Epidemiology and Public Health (CIBERESP), Barcelona, Spain; 2 Cardiovascular Epidemiology & Genetics group, Inflammatory and Cardiovascular Disease Programme, Institut Municipal d'Investigaci/'o Mèdica (IMIM), Barcelona, Spain; 3 Statistics Department, University of Barcelona (UB), Barcelona, Spain; 4 Structural Biology and Biocomputing Programme, Spanish National Cancer Centre (CNIO), Madrid, Spain; 5 Center for Research in Environmental Epidemiology (CREAL), Barcelona, Spain

## Abstract

**Background:**

Copy number variants (CNV) are a potentially important component of the genetic contribution to risk of common complex diseases. Analysis of the association between CNVs and disease requires that uncertainty in CNV copy-number calls, which can be substantial, be taken into account; failure to consider this uncertainty can lead to biased results. Therefore, there is a need to develop and use appropriate statistical tools. To address this issue, we have developed CNVassoc, an R package for carrying out association analysis of common copy number variants in population-based studies. This package includes functions for testing for association with different classes of response variables (e.g. class status, censored data, counts) under a series of study designs (case-control, cohort, etc) and inheritance models, adjusting for covariates. The package includes functions for inferring copy number (CNV genotype calling), but can also accept copy number data generated by other algorithms (e.g. CANARY, CGHcall, IMPUTE).

**Results:**

Here we present a new R package, CNVassoc, that can deal with different types of CNV arising from different platforms such as MLPA o aCGH. Through a real data example we illustrate that our method is able to incorporate uncertainty in the association process. We also show how our package can also be useful when analyzing imputed data when analyzing imputed SNPs. Through a simulation study we show that CNVassoc outperforms CNVtools in terms of computing time as well as in convergence failure rate.

**Conclusions:**

We provide a package that outperforms the existing ones in terms of modelling flexibility, power, convergence rate, ease of covariate adjustment, and requirements for sample size and signal quality. Therefore, we offer CNVassoc as a method for routine use in CNV association studies.

## Background

The proportion of variation in risk of complex diseases explained by the single nucleotide polymorphisms (SNPs) that have been discovered in recent years using the genome-wide association approach appears to limited. This has lead to the suggestion that other, possibly more complex, genetic variants could partly explain the remaining disease susceptibility. Technological advances now allow a class of genetic variants known as copy number variants (CNV) to be genotyped with increasing levels of accuracy, and several studies have recently explored the relationship between these variants and risk of complex disease [1,2]. Genotyping these kinds of complex genetic markers is still a challenge and current laboratory techniques and platforms often contain a non-negligible percentage of errors. In order to minimise bias in the results of association studies involving CNVs, uncertainty in these copy number calls must be taken into account in the analysis. In addition, large-scale CNV genotyping projects need a tool to automate the analysis of thousands of CNVs. Here, we present CNVassoc, an R package [3] designed to analyze CNV data. Methodological details of the algorithms and applications implemented in CNVassoc are described in [4]. In addition to these, other techniques, such as accounting for batch effects in inferring copy number status, or modelling other response distributions (Poisson or Weibull for censored data) have now been incorporated into CNVassoc. In this application note we present an overview of the package. The Additional file 1 contains a tutorial (the vignette for the package) together with technical notes on the derivation of the likelihoods for the different models.

## Implementation

We developed a set of functions to analyse copy number variants and integrated them as an R package called CNVassoc. Also, we created a very extensive manual of the package (vignette) with several examples of real and simulated data explaining how to use the package functions and their capabilities.

The R software is a general purpose and open source program commonly used in all type of statistical analysis. Having incorporated the functions as an R package allows user to take advantage of R flexibility in manipulating the input and the results when analysing CNVs with CNVassoc. In addition, we structured CNVassoc functions and results in methods and classes to make the package usage easier and more intuitive.

### Software main features

To date, only one other R package, CNVtools[5], has been developed that can appropriately incorporate CNV copy number call uncertainty in the test for association between CNVs and disease. However, CNVtools has some limitations, mainly related to the fact that the copy number calling and association testing steps are combined in a single procedure. The current version of CNVtoolshttp://bioconductor.org uses complex and computationally intensive algorithms, cannot adjust for covariates, and can only model binary and normally distributed responses. By separating these two steps, CNVassoc offers significant advances in terms of analytical flexibility and computational speed.

### Inferring copy number status

By separating the CNV calling and association testing steps, CNVassoc allows the user to test for association between CNVs and disease using copy number probabilities from any source. While the use of probability data from more powerful calling algorithms such as CGHcall [6], IMPUTE [7,8] or CANARY [9] is recommended, CNVassoc provides several tools for inferring copy number status, where necessary. For example, CNVassoc can fit a mixture of normal distributions to CNV signal intensity data [10], or assign copy number status by defining a set of signal intensity cut points, which might be useful when analysing probe intensity data from MLPA [11] or qPCR [12]. In addition, there is an option to take batch effects into account, in order to reduce false positives and provide robust estimates, as discussed in [5].

#### Considering batch effect

In CNVassoc, the batch effect has been handled in the following way:

Formally, the intensity signal distribution, *y*, is supposed to follow a mixture of gaussian distributions,

where, *ϕ *is the gaussian density function, *μ_cb _*and *σ_cb _*is the mean and standard deviation respectively of intensity signal for *c *copy number variants in *b*-th batch, and *w_c _*is the proportion of individuals with *c *copies in the population. Notice that mean and standard deviation can vary not only between copy number status but also between batches, but the copy number status prevalences (*w_c_*) not. If *μ_cb _*and *σ_cb _*varies between batches and batches are associated with the disease/response, then the batch effect exists by definition, and can lead to false association if it is not taken into account [5].

In CNVassoc, specific means, standard deviations and prevalences estimates are calculated separately using data from each batch. Then, prevalences estimates are obtained averaging from specific prevalences:

where *n_b _*is the number of sample individuals in the *b*-th batch, *B *is the total number of batches in the sample, and *n *is the total number of individuals in the sample.

### Improved association test

To incorporate CNV copy number uncertainty in the association test, CNVassoc uses a simpler model formulation than that of CNVtools. This allows us to use the faster Newton-Raphson procedure, which yields not only the effect estimate for the CNV, but also its confidence interval.

### Adjustment for covariates

CNVassoc can fit association models adjusted for covariates (age, gender, smoking, etc.), which may be particularly important where it is necessary to adjust for population stratification [13].

### Response phenotypes

CNVassoc can be used to analyse dichotomous (Binomial), count (Poisson), or continuous (Gaussian) response phenotypes, as well as data from cohort studies (Weibull).

### Inheritance models

CNVassoc can perform association analysis under a codominant (additive) model, which assumes a constant effect on phenotype per unit change in copy number, or under a model-free design, which treats each copy number as an independent category.

### Analysis of multiple CNVs

To perform association testing of multiple CNVs with greater computational efficiency, a function called multiCNVassoc has been implemented. When multiple processors are available, it can parallelize association tests using the Snow package http://www.sfu.ca/~sblay/R/snow.html. An example of association tests involving several CNVs is shown in Section 3 of the Additional file 1 where data from a CGH array is analysed.

### Computational Efficiency

Using the same sample sizes and probe signal intensity distributions as used in [5], we performed a simulation study in order to compare the performance of the methods implemented in CNVassoc and CNVtools. We observed that both methods performed well, but we note that CNVassoc has a number of important advantages over CNVtools in terms of computational speed and robustness in situations of limited sample sizes.

### Performing association tests

First, an object of class cnv must be created by CNVassoc or using probabilities from other algorithms. Then, an association test between the CNV and disease can be performed using the CNVassoc function, which returns an object of class 'CNVassoc'. Associated print and summary functions give exhaustive outputs. The (CNVtest) function computes an overall p-value to test whether a CNV is associated with the disease

### Functions to simulate CNV data

In CNVassoc package, function to simulate CNV data have been implemented. It is possible to simulate data from different type of responses and studies: case-control (simCNVdataCaseCon), cohort with binary response (simCNVdataBinary), counting process with poisson-distributed response (simCNVdataPois), quantitative normal-distributed response (simCNVdataNorm) and time-to-event with right-censored-weibull-distributed response (simCNVdataWeibull).

### Association analysis on imputed SNPs

Also, it is possible to analyse association of imputed SNPs and response. Taking the genotypes probabilities obtained from any software capable to impute SNPs, such as IMPUTE [7,8], association analysis for case-control studies, cohort, quantitative or counting response can be performed with CNVassoc. In section 5 of the Additional file 1 we show in detail how to analyse a data set downloadable from SNPTEST website which contains probabilities of different imputed genotypes from different SNPs among a set of cases and controls.

## Results and Discussion

In this section we show the results obtained in inferring copy number status and association analysis on a real data set including 360 cases and 291 controls (data described in [4]). The data contains peaks intensities for two genes arising from an MLPA assay. From this example, we present the main CNVassoc functions and illustrate how to use them to infer copy number copies and estimate association on case-control status.

A more detailed description of all these analyses and others (imputed SNPs, aCGH data, other phenotypes distributions -poisson, weibull and normal-) can be found in Additional file 1.

### Inferring copy number status

Previous to association analysis, inferring copy number status process must be done. To do so, the function cnv is used. In this subsection, gene 2 from MLPA data example is used. This data set can be load from the CNVassoc package.

>*library(CNVassoc)*

>*data(dataMLPA)*

>*CNV <- cnv(x = dataMLPA$Gene2, threshold.0 = 0.01, mix.method = "mixdist")*

**Figure 1:**
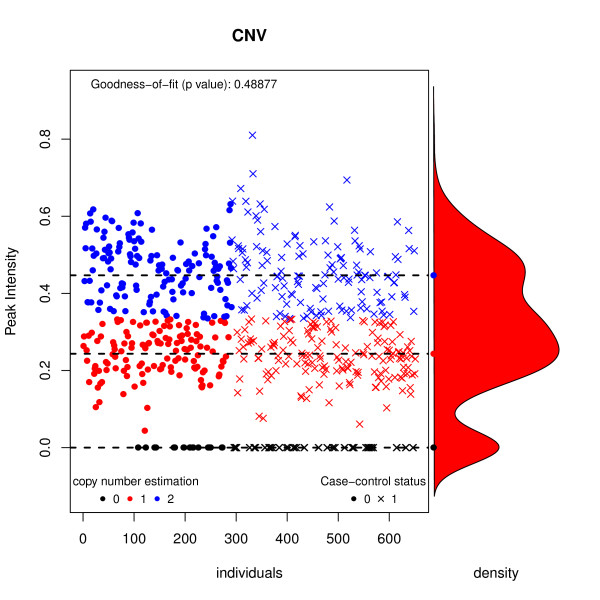
**Plot of a cnv object generated from CNV signal intensity data**.

The peak intensities of gene 2 are assumed to follow a mixture of normal distributions, and the method used to estimate this distribution is specified by the mix.method argument. When threshold.0 = 0.01, all individuals with peak intensities lower than 0.01 are assumed to carry 0 copies. The CNV object is of class cnv, which can be printed and plotted (Figure 1).

>*CNV*

Inferred copy number variant by a quantitative signal

   Method: function mix {package: mixdist}

-. Number of individuals: 651

-. Copies 0, 1, 2

-. Estimated means: 0, 0.2435, 0.4469

-. Estimated variances: 0, 0.0041, 0.0095

-. Estimated proportions: 0.1306, 0.4187, 0.4507

-. Goodness-of-fit test: p-value = 0.4887659

-. Note: number of classes has been selected using the best BIC

>*plot(CNV)*

A measure that quantifies the amount of uncertainty in the CNV calling estimation can be computed using the function getQualityScore. Various measures are available; the following is an example of how to obtain the quality score (uncertainty measure) described in the CNVtools paper [5]:

>*getQualityScore(CNV, type = "CNVtools")*

--CNVtools Quality Score: 3.057171

In some cases, it may be preferable to infer copy number status using another algorithm that is not implemented in CNVassoc, e.g. if the probe signal intensities do not follow a mixture of normal distributions. A matrix of copy number probabilities obtained from other algorithms can be used as input for the cnv function to create a cnv class object, which can then be used to perform association analysis. Also, it is possible to take suspected batch effects in the signal intensity distributions into account by specifying the batch variable using the batch argument in the cnv function. This is important in order to avoid false positives in the posterior association model estimation, as suggested in [5]. A more detailed explanation and example of this issue can be found in section 4.2 of Additional File 1.

### Performing association models

To carry out association analysis between CNV and disease, the function CNVassoc is used. This function incorporates copy number call uncertainty by using a latent class model as described in [4]. The response variable (disease) can be: binary, quantitative (normally distributed), from a counting process, time to event (Weibull distributed). Also, an additive or model-free pattern of inheritance can be analysed. The result returned by the CNVassoc function is an object that can be printed and summarized and its structure is very similar to other well known R functions such as glm.

Here, we continue with the same MLPA data taking the CNV object for gene 2 in the previous section. To fit a logistic regression model with case-control status as a response and CNV copy number as a predictor, and assuming an additive genetic effect, we type

>*mod <- CNVassoc(casco ~ CNV, data = dataMLPA)*

>*summary(mod)*

Call:

CNVassoc(formula = casco ~ CNV, data = dataMLPA)

Deviance: 876.396

Number of parameters: 3

Number of individuals: 651

Coefficients:

           OR lower.lim upper.lim     SE      Stat    pvalue

CNV0   1.0000

CNV1   0.4772   0.2742   0.8304   0.2827   -2.6172      0.009

CNV2   0.3169   0.1834   0.5477   0.2791   -4.1169   3.84e-05

(Dispersion parameter for binomial family taken to be 1)

Covariance between coefficients:

     CNV0     CNV1     CNV2

CNV0   0.0613  0.0000   0.0000

CNV1           0.0186  -0.0032

CNV2                    0.0166

By applying the summary function to the result, we obtain odds ratios, confidence intervals, and p-values for every copy number status with respect to the reference copy number category.

To compute the global CNV significance p-value, the CNVtest function can be used as follows:

>*CNVtest(mod, "LRT")*

----CNV Likelihood Ratio Test----

Chi = 18.75453 (df = 2), pvalue = 8.462633e-05

In this example, a Likelihood Ratio Test (LRT) is computed, comparing a model containing CNV to a model lacking CNV (i.e. a model without predictors or the null model).

Using the CNVassoc function it is possible to change the inheritance model to additive (changing the model argument), or adjust for other covariates (such as age, sex, or principal components) in the formula argument in the usual way. Also, other types of response can be analysed changing the family argument. More detailed examples are in the Additional file 1.

### Response phenotypes: Weibull

In this section, we illustrate how to analyse a time-to-event response variable (Weibull distributed) using simulated data generated with the function simCNVdataWeibull. In the following example, a CNV has been generated with 0, 1 and 2 possible copies with probabilities of 25%, 50% and 25% respectively, with intensity signal standard deviation of 0.4 for each copy status, and means of 0, 1 and 2 respectively. The response variable has been simulated under a Weibull distribution with shape parameter equal to 1 and disease incidence equal to 0.05 (per person-year) among the population with zero copies (reference). The proportion of observed events (non-censored) was set to 10%. Finally, these data have been generated assuming a additive CNV effect with a Hazard Ratio of 1.5 per copy.

>*set.seed(123456)*

>*n <- 5000*

>*w <- c(0.25, 0.5, 0.25)*

>*mu.surrog <- 0:2*

>*sd.surrog <- rep(0.4, 3)*

>*hr <- 1.5*

>*incid0 <- 0.05*

>lambda <- c(incid0, incid0 * hr, incid0 * hr^2)

>*shape <- 1*

>*scale <- lambda^(-1/shape)*

>*perc.obs <- 0.1*

>*time.cens <- qweibull(perc.obs, mean(shape), mean(scale))*

>*dsim <- simCNVdataWeibull(n, mu.surrog, sd.surrog, w, lambda,*

+   *shape, time.cens)*

Once the CNV data and phenotype has been generated, inferring copy number status and fitting the association model is performed in the following two steps:

(1) Inferring copy number status, as for case-control studies:

>*CNV <- cnv(dsim$surrog, mix = "mclust")*

>*attr(CNV, "num.copies") <- 0:2*

Note that 3 copy number statuses has been estimated by BIC criteria. By default 1, 2 and 3 copies are assigned. The number of copies for each status can be changed to 0, 1 and 2 respectively by modifying the num.copies attribute.

2) Testing for association between CNV and time-to-event, specifying the family argument as "weibull":

>*fit <- CNVassoc(Surv(resp, cens) ~ CNV, data = dsim, family = "weibull",*

+   *model = "add")*

>*coef(summary(fit))*

                  HR lower.lim  upper.lim            SE      stat         pvalue

    trend   1.385556   1.205619   1.592348   0.07097498   4.594595   4.335896e-06

Note that, Hazard Ratios (HR) are displayed instead of Odds Ratios. In this case, an additive CNV effect has been assumed in performing the association model.

### Computational Efficiency

In this section, we compare the performance of CNVassoc in terms of speed and convergence rate to that of CNVtools, which is the only other tool that is currently available for performing CNV association analysis, while correctly taking copy number uncertainty into account. Simulated case-control data was generated for different sample sizes (500 cases and 500 controls; 2,000 cases and 2,000 controls), and different degrees of call uncertainty, from very little uncertainty (*Q *= 6) to a moderate-high degree of uncertainty (*Q *= 3). A single CNV marker has been simulated using 1,000 iterations (simulations), under the described scenarios. In each simulation, univariate probe signal intensities (similar to MLPA) have been generated from a gaussian mixture distribution, and copy number status has been inferred from them. After this, an association model has been performed using the proposed method (Latent Class model). The uncertainty measure, *Q*, was proposed by [5] (see page 3); values of *Q *below 3 indicate moderate-high uncertainty and this must be taking into account in the association analysis, while values of *Q *bigger than 4.5 or 5 indicate that uncertainty is almost insignificant. Table 1 shows the number of times model estimation fails using CNVassoc and CNVtools under these various scenarios. CNVassoc converges in all simulations, except when sample size is small and uncertainty is high. When sample size is high (2,000 cases and 2,000 controls) CNVassoc converges in all situations, while CNVtools fails in some simulations when uncertainty is high. And when sample size is moderate-low (500 cases and 500 controls), CNVassoc converges almost in all times except when uncertainty is high (*Q *< 3.5), while CNVtools fails in some simulations even when the degree of uncertainty is low (*Q *= 6) and starts to fail in the majority of situations when uncertainty is moderate (*Q *< 4) and performs even worse when is high.

**Table 1:** Number of failed convergence simulations out of 500 using CNVassoc and CNVtools according to inferring copy number uncertainty *Q *and number of cases *N*.

	*N *= 2000		*N *= 500	
		
*Q*	CNVassoc	CNVtools	CNVassoc	CNVtools
6.0	0	0	0	15
5.5	0	0	0	20
5.0	0	0	0	65
4.5	0	0	0	92
4.2	0	0	0	187
4.0	0	0	0	246
3.7	0	0	0	294
3.5	0	1	0	299
3.2	0	13	212	389
3.0	0	65	331	400

We have also observed a marked difference in the speed of each procedure: when analyzing 10,000 CNVs in 2,000 cases and 2,000 controls, and with a *Q *= 4, CNVtools took 1 day and 17 hours to complete the analysis, whereas CNVassoc took just 90 minutes; with *Q *= 3, CNVtools took 6 days and 16 hours, but CNVassoc took only 2 hours. More comparisons between CNVassoc and CNVtools are shown in section 4.3.1 of Additional file 1.

## Conclusions

We present a new package for performing analysis of association between copy number variants and disease, appropriately taking uncertainty in CNV copy number calls into account. The numerical procedure for fitting the model is simple and computationally efficient, handling thousands of CNVs in reasonable time. In addition, it is possible to adjust for covariates which may be necessary to control for population stratification. A central feature of CNVassoc is that input data can come from any CNV calling algorithm that produces copy number probabilities. Note that the CNVassoc package can also be applied to SNPs. For instance, in the context of imputed SNPs (e.g., IMPUTE [7,8], BIMBAM [14], MACH1 http://www.sph.umich.edu/csg/abecasis/MACH/, etc.) the probability estimates of each genotype coming from this software can easily be incorporated to our functions. We intend to continue developing the package, and expect to incorporate CNV * non-genetic predictor interactions, and CNV * CNV interactions, in the near future.

In conclusion, considering the advantages in terms of modelling flexibility, power, convergence rate, ease of covariate adjustment, and requirements for sample size and signal quality, we offer CNVassoc as a method for routine use in CNV association studies.

## Availability and requirements

1. Project name: CNVassoc

2. Project home page: http://www.creal.cat/jrgonzalez/software.htm and http://www.cran.r-project.org

3. Operating system(s): Platform independent

4. Programming language: R

5. R Dependencies: mixdist, mclust, survival

6. R Suggested: CGHcall, CGHregions, snow, CNVtools

7. License: GPL or newer

## Competing interests

The authors declare that they have no competing interests.

## Authors' contributions

JRG conceived the idea of incorporation probabilities to address uncertainty in CNV association studies. IS and JRG created the R functions and the package. IS implemented some R functions to simulate CNV data. GL drafted the manuscript. IS, GL, RD-U and JRG designed, performed and interpreted the simulation studies to compare CNVtools and CNVassoc. IS, RD-U and JRG helped to draft the manuscript. All authors read and approved the final manuscript.

## Pre-publication history

The pre-publication history for this paper can be accessed here:

http://www.biomedcentral.com/1755-8794/4/47/prepub

## Supplementary Material

Additional file 1 **User's manual**. CNVassoc_manual.pdf is the user's guide of CNVassoc package, where detailed examples with real and simulated data are shown, illustrating how to use the CNVassoc package functions. Click here for file
