# Supplementary material for: CNVassoc: Association analysis of CNV data using R
Source: BMC Med Genomics. 2011 May 24;4:47. doi: 10.1186/1755-8794-4-47 (PMC3121578; doi:10.1186/1755-8794-4-47)
Supplement: Additional file 1 — User's manual. CNVassoc_manual.pdf is the user's guide of CNVassoc package, where detailed examples with real and simulated data are shown, illustrating how to use the CNVassoc package functions. [file 1755-8794-4-47-S1.PDF]

CNVassoc: Association analysis of CNV data

Isaac Subirana[1,2,3], Ramon Diaz-Uriarte[4], Gavin Lucas[2], Juan-Ramon Gonzalez[5,1]

March 29, 2011

[1]CIBER Epidemiology and Public Health (CIBERESP), Spain
[2]Cardiovascular Epidemiology & Genetics group, Inflammatory and Cardiovascular Disease Programme, IMIM, Hospital del Mar Research Institute, Spain
[3]Statistics Department, University of Barcelona, Spain
[4]Spanish National Cancer Centre (CNIO), Spain
[5]Center for Research in Environmental Epidemiology (CREAL), Spain

jrgonzalez@creal.cat http://www.creal.cat/jrgonzalez/software.htm

Contents

1 Introduction **2**

2 CNV from a single probe **2**
2.1 The data 2
2.2 Inferring copy number status from signal data 7
2.2.1 From univariate signal intensity 7
2.2.2 From other algorithms 7
2.2.3 From predetermined thresholds 8
2.3 Summarizing information 8
2.4 Measuring uncertainty in inferring copy number status 11
2.5 Assessing associations between CNV and disease 12
2.5.1 Modelling association 12
2.5.2 Testing associations 15
2.6 Analysing other genetic models 17

3 CNV from aCGH **19**

4 Illumina data **22**
4.1 Preparing signal data 23
4.2 Inferring copy number status considering batch effect 23
4.3 Association model: comparison with results from `CNVtools` 26
4.3.1 Power and computation time of CNVassoc and CNVtools 26

5 Imputed data (SNPTEST format) **29**

6 Other phenotype distributions **31**
6.1 Poisson distributed phenotype 31
6.2 Weibull distributed phenotype 34

1 Introduction

CNVassoc allows users to perform association analysis between CNVs and disease incorporating uncertainty of CNV genotype. This document provides an overview on the usage of the CNVassoc package. For more detailed information on the model and assumption please refer to article [3] and its supplementary material. We illustrate how to analyze CNV data by using some real data sets. The first data set belongs to a case-control study where peak intensities from MLPA assays were obtained for two different genes. The second example corresponds to the Neve dataset [6] that is available at Bioconductor. The data consists of 50 CGH arrays of 1MB resolution for patients diagnosed with breast cancer. All datasets are available directly from the CNVassoc package. Finally, we show examples with Poisson and Weibull-distributed phenotypes

Start by loading the package CNVassoc:

```
> library(CNVassoc)
```

and some required libraries

```
> library(xtable)
```

2 CNV from a single probe

2.1 The data

In order to illustrate how to assess association between CNV and disease, we use a data set including 360 cases and 291 controls. Data is to be published soon as described in [3]. The data contains peaks intensities for two genes arising from an MLPA assay. Note that Illumina or Affymetrix data, where log2 ratios are available instead of peak intensities, can be analyzed in the same way as we are illustrating.

The MLPA data set contains case control status as well as two simulated covariates (quanti and cov) that have been generated for illustrative purposes (e.g., association between a quantitative trait and CNV or how to adjust for covariates). To load the MLPA data just type

```
> data(dataMLPA)
> head(dataMLPA)

    id casco Gene1     Gene2 PCR.Gene1 PCR.Gene2 quanti   cov
1 H238     1  0.51 0.5385080        wt        wt  -0.61 10.83
2 H238     1  0.45 0.6392029        wt        wt  -0.13 10.69
3 H239     1  0.00 0.4831572       del        wt  -0.57  9.63
4 H239     1  0.00 0.4640072       del        wt  -1.40  9.87
5 H276     1  0.00 0.0000000       del       del   0.83 10.25
6 H276     1  0.00 0.0000000       del       del  -2.07 10.40
```

First, we look at the distribution of peak intensities for each of the two genes analyzed: see Figure 1.

Figure 1 shows the signals for Gene 1 and Gene 2. For both genes it is clear that there are 3 clusters corresponding to 0, 1 and 2 copies. However, the three peaks for Gene 2 are not so well separated as those of Gene 1 (the underlying distributions overlap much more). This fact leads to more uncertainty when inferring the copy number status for each individual. This will be illustrated in the next section.

In the CNVassoc package, a function called plotSignal has been implemented to plot the peak intensities for a gene. To illustrate this, a plot of the intensities of Gene 2 for each individual, distinguishing between cases and controls, can be performed by typing (see figure 2)

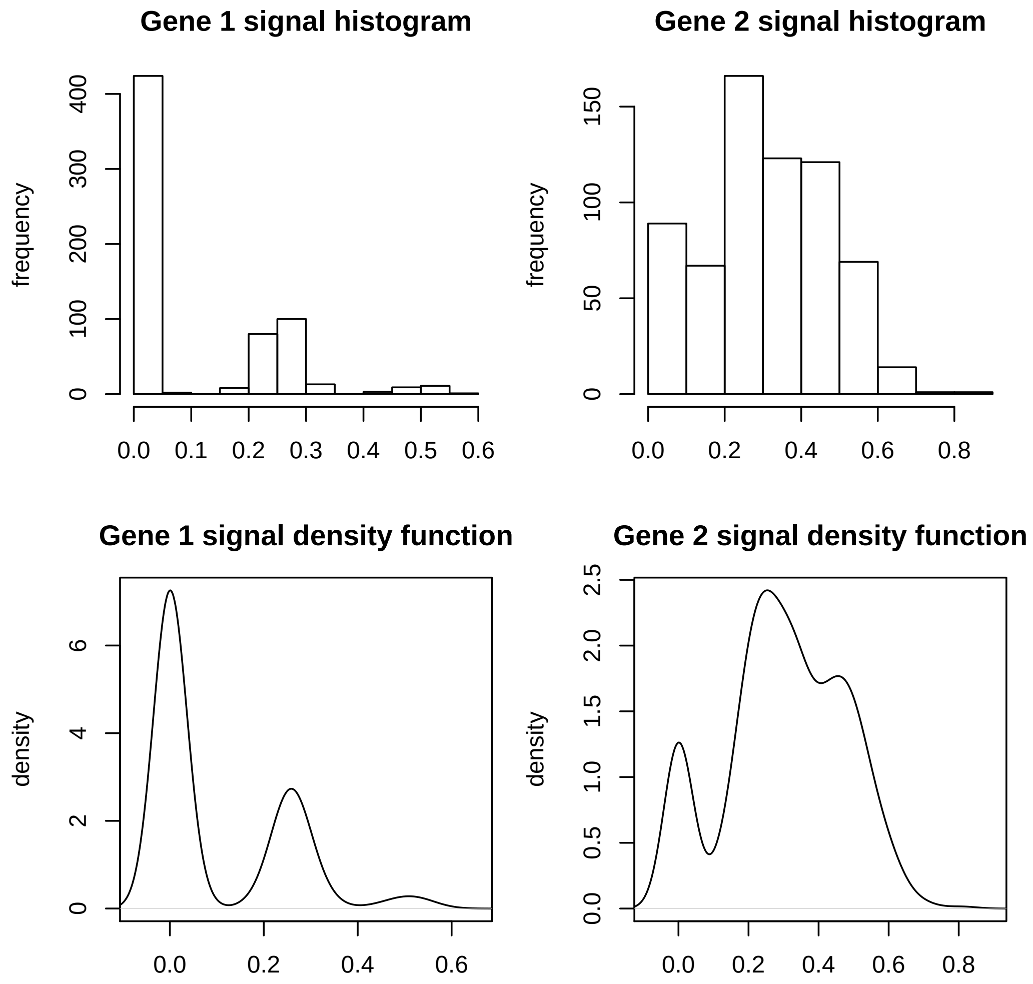

Figure 1: Signal distributions for Gene 1 and Gene 2

```
> plotSignal(dataMLPA$Gene2, case.control = dataMLPA$casco)
```

or, similarly but correlating the peak intensities with a quantitative phenotype (see figure 3) type

```
> plotSignal(dataMLPA$Gene2, case.control = dataMLPA$quanti)
```

In figure 3, the quantitative phenotype is plotted on the x-axis, instead of distinguishing points by shape, as in figure 2.

Also, it is possible to specify the number of cutoff points and place them interactively via `locator` on the previous plot, in order to infer the copy number status in a naive way. (More sophisticated ways of inferring copy number status will be dealt with in subsequent sections). To place 2 cutoff points, thereby defining 3 copy number status values or clusters, (note use of argument `n=2`) and store them as `cutpoints`:

```
\dontrun{
cutpoints<-plotSignal(dataMLPA$Gene2,case.control=dataMLPA$casco,n=2)
}
```

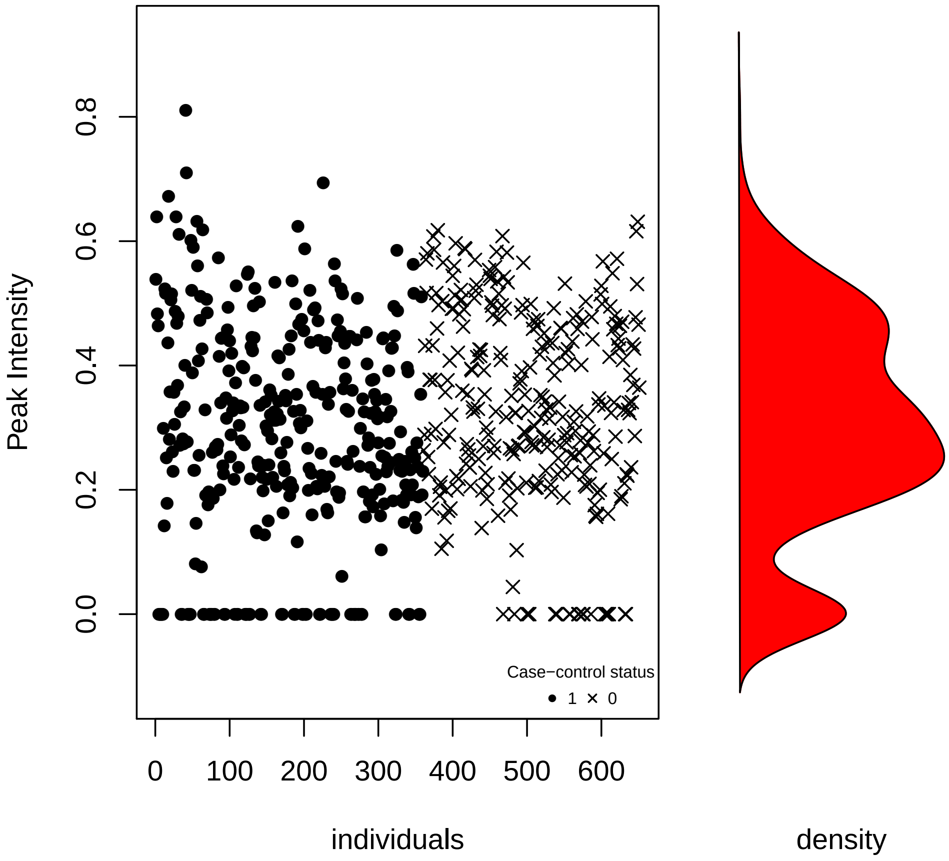

Figure 2: Signal distribution for Gene 2 using `plotSignal`

The plot generated in figure 4, is similar to that of 2, but using colours to distinguish copy number status values inferred from the cutoff points.

In this example, the cutoff points have been placed at:

```
> cutpoints
```

```
[1] 0.08470221 0.40485249
```

These stored cutoff points will be used in the following sections.

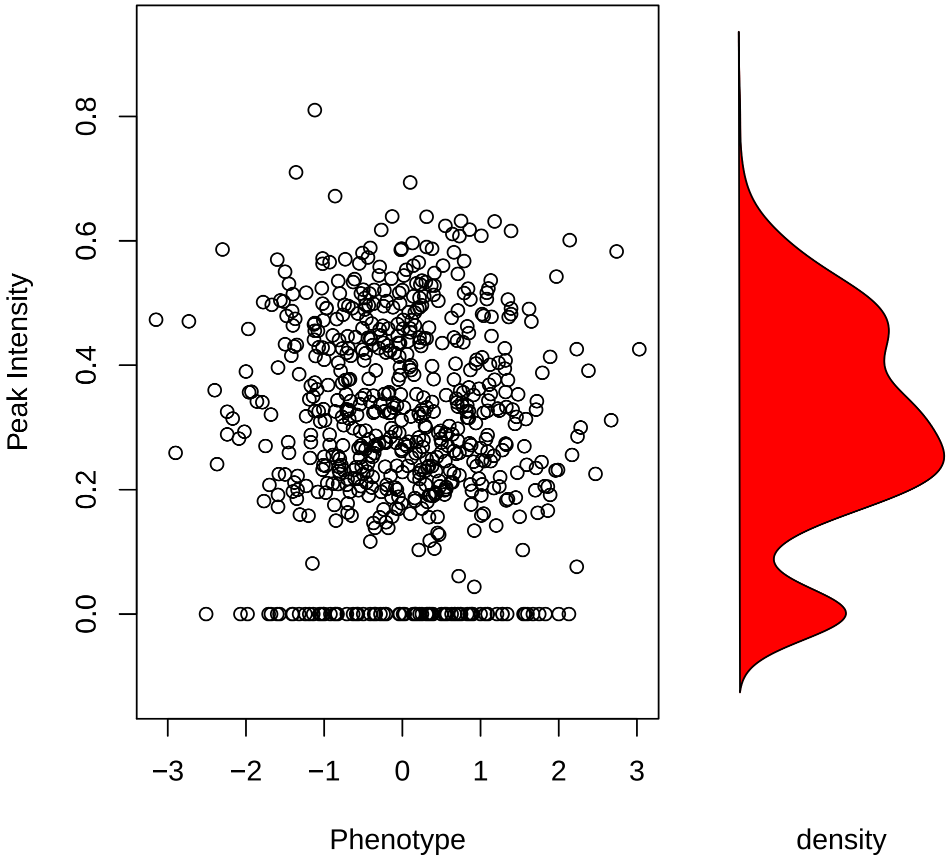

Figure 3: Signal distribution for Gene 2 using `plotSignal`

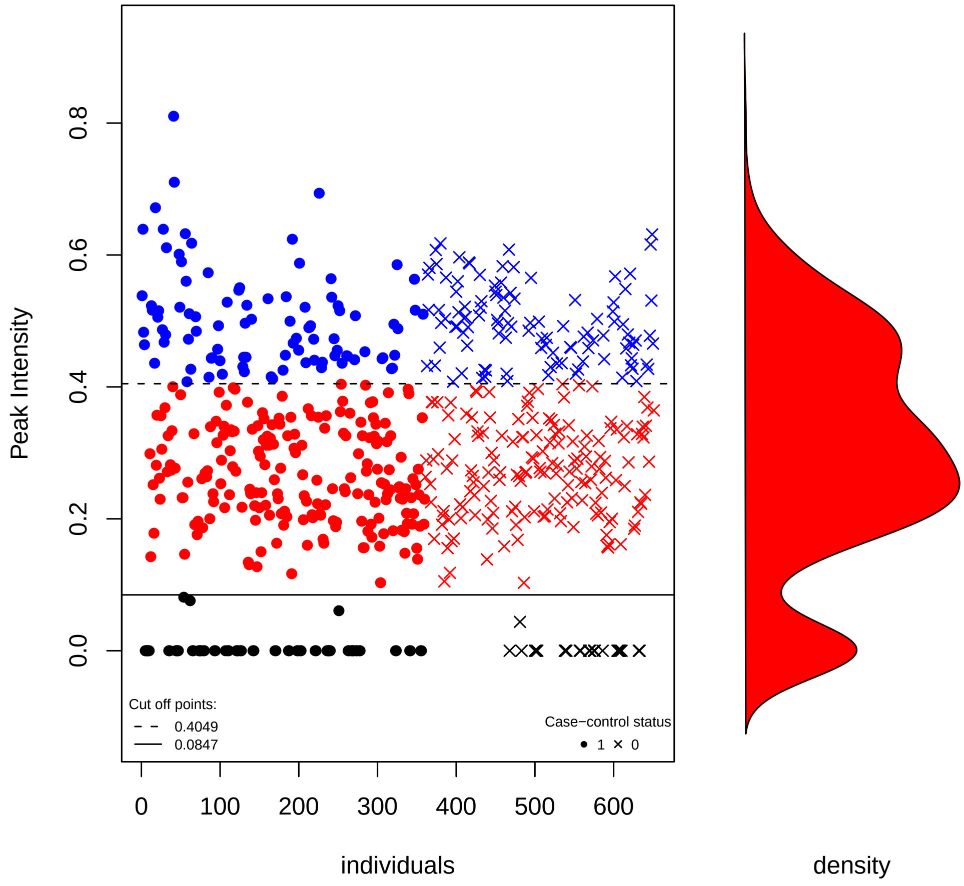

Figure 4: Signal distribution for Gene 2 using `plotSignal` once cutoff points have been set with `locator`

2.2 Inferring copy number status from signal data

2.2.1 From univariate signal intensity

The cnv function is used to infer the copy number status for each subject using the quantitative signal for an individual probe. This signal can be obtained from any platform (MLPA, Illumina, ...).

This function assumes a normal mixture model as other authors have proposed in the context of aCGH [7, 9]. It should be pointed out that in some instances, the intensity distributions (see Gene 1 in Figure 1) for a null allele are expected to be equal to 0. Due to experimental noise these intensities can deviate slightly from this theoretical value. For these cases, the normal mixture model fails because the underlying distribution of individuals with 0 copies is not normal. In these situations we fit a modified mixture model (see [3] for further details).

Figure 1 presents two distinctly different scenarios. For Gene 1 there are clearly three different status values, but for Gene 2 the situation is not so clear.

Function cnv provides various arguments to cope with all these issues. The calling for Gene 1 can be done by executing

```
> CNV.1 <- cnv(x = dataMLPA$Gene1, threshold.0 = 0.06, num.class = 3,
+     mix.method = "mixdist")
```

The argument threshold.0=0.06 indicates that individuals with peak intensities lower than 0.06 will have 0 copies. Since there are three underlying copy number status values, we set argument num.class to 3. Argument mix.method indicates what algorithm to use in estimating the normal mixture model. "mixdist" uses a combination of a Newton-type method and the EM algorithm implemented in the mixdist library, while "mclust" uses the EM algotithm implemented in the Mclust library.

When the exact number of components for the mixture model is unknown (which may be the case for Gene 2), the function uses the Bayesian Information Criteria (BIC) to select the number of components. This is performed when the argument num.class is missing. In this case the function estimates the mixture model admitting from 2 up to 6 copy number status values.

```
> CNV.2 <- cnv(x = dataMLPA$Gene2, threshold.0 = 0.01, mix.method = "mixdist")
```

As we can see, the best model has a copy number status of 3. This result, obtained by using BIC, is as expected because we already know that this gene has 0, 1 and 2 copies (see [3]).

2.2.2 From other algorithms

The result of applying function cnv is an object of class cnv that, among other things, contains the posterior probabilities matrix for each individual. This information is then used in the association analysis where the uncertainty is taken into account. Posterior probabilities from any other calling algorithms can also be encapsulated in a cnv object to be further used in the analysis.

To illustrate this, we will use the posterior probability matrix that has been computed when inferring copy number for Gene 2 by using the normal mixture model. This information is saved as an attribute for an object of class cnv. A function called getProbs has been implemented to simplify accessing this attribute. Thus the probability matrix can be saved in an object probs.2 like this:

```
> probs.2 <- getProbs(CNV.2)
```

Imagine that probs.2 contains posterior probabilities obtained from some calling algorithm such as CANARY (from PLINK) or GCHcall (this will be further illustrated in Section 3). In this case, we create the object of class cnv that will be used in the association step by typing

```
> CNV.2probs <- cnv(probs.2)
```

2.2.3 From predetermined thresholds

Inferring copy number status for Gene 2 from previously specified threshold points (stored in vector `cutpoints`) can be done using the same `cnv` function but setting the argument `cutoffs` to `cutpoints`.

```
> CNV.2th <- cnv(x = dataMLPA$Gene2, cutoffs = cutpoints)
```

Now, the inferred copy number object `CNV.2th` contains the same information as it would if it had been created directly from probabilities.

2.3 Summarizing information

We have implemented two generic functions for an object of class `cnv`. The generic `print` function gives the results on inferred copy number status. It includes the means, variances and proportions of copy number clusters as well as the p value corresponding to the goodness-of-fit test for the selected number of classes.

```
> CNV.1

Inferred copy number variant by a quantitative signal
   Method: function mix {package: mixdist}

-. Number of individuals: 651
-. Copies 0, 1, 2
-. Estimated means: 0, 0.2543, 0.4958
-. Estimated variances: 0, 9e-04, 0.0012
-. Estimated proportions: 0.6544, 0.3088, 0.0369
-. Goodness-of-fit test: p-value= 0.6615318
```

and for Gene 2

```
> CNV.2

Inferred copy number variant by a quantitative signal
   Method: function mix {package: mixdist}

-. Number of individuals: 651
-. Copies 0, 1, 2
-. Estimated means: 0, 0.2435, 0.4469
-. Estimated variances: 0, 0.0041, 0.0095
-. Estimated proportions: 0.1306, 0.4187, 0.4507
-. Goodness-of-fit test: p-value= 0.4887659


-. Note: number of classes has been selected using the best BIC
```

This report differs slightly when the object was created from only posterior probabilities:

```
> CNV.2probs
```

```
-. Copy number variant
   Input data: called probabilities
-. Number of individuals: 651
-. Copies 0, 1, 2
-. Estimated proportions: 0.1306, 0.4187, 0.4507
```

Figure 5 shows the result of invoking the generic `plot` function on these objects.

```
> pdf("./figures/fig2a.pdf")
> plot(CNV.1, case.control = dataMLPA$casco, main = "Gene 1")
> dev.off()

windows
      2

> pdf("./figures/fig2b.pdf")
> plot(CNV.2, case.control = dataMLPA$casco, main = "Gene 2")
> dev.off()

windows
      2
```

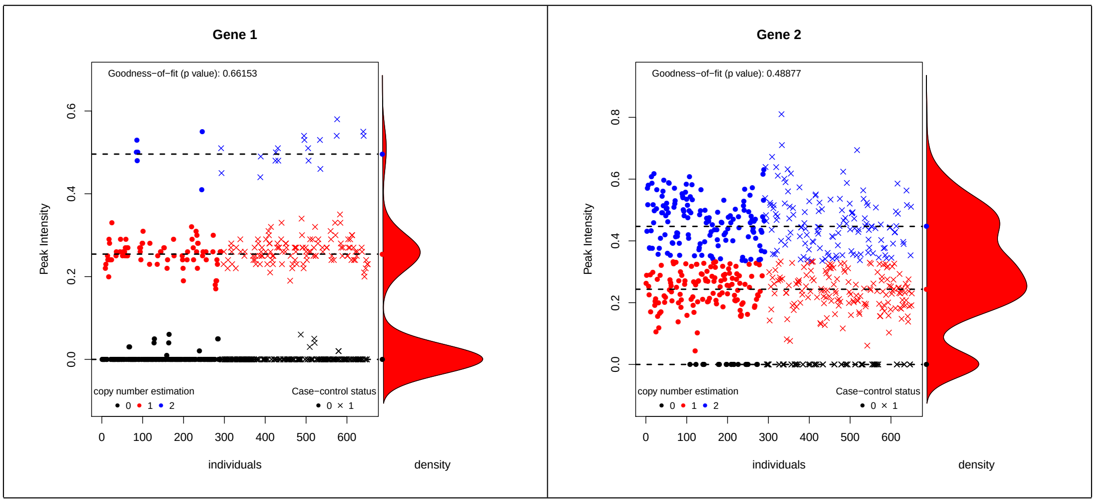

Figure 5: Signal distribution by case control, and inferred number of copies

In figure 5 the signal is coloured by the inferred (most probable) copy number, while cases and controls are distinguished by shape. This last option is specified by the argument `case.control`. On the right side of the plot, a density function of signal distribution is drawn. The p-value of goodness-of-fit test is the same as this described in the beginning of this section. It indicates whether the assumed normal mixture model (with a given number of components) is correct or not. Notice that for both genes the intensity data fits our the model well (goodness-of-fit p-values > 0.1).

The action of `plot` when only posterior probabilities are available gives a different result (Figure 6). Two barplots are created for cases and controls (when argument `case.control` is used). Both are split by the copy number frequency.

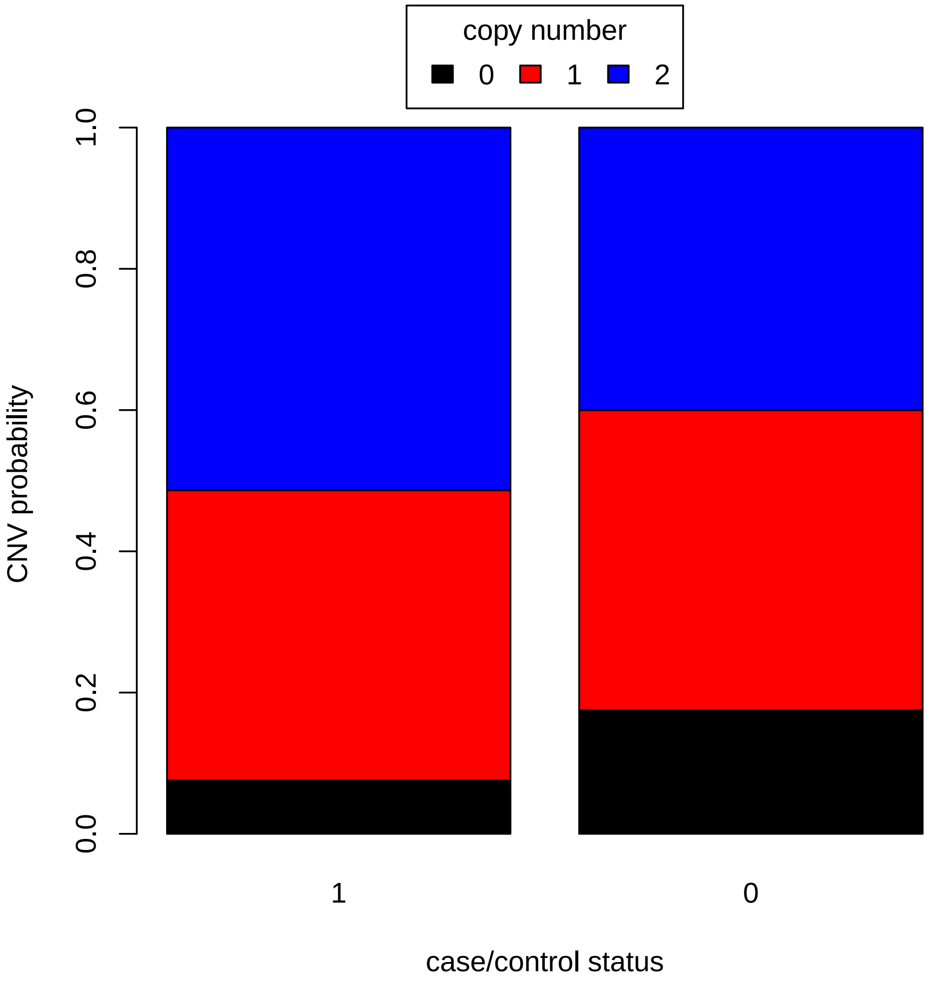

Figure 6: Estimated copy number frequencies for Gene 1 and Gene 2

2.4 Measuring uncertainty in inferring copy number status

The function `getQualityScore` uses information from an object of class `cnv` to compute a value that indicates how much the underlying copy number distribution (peak intensities) are mixed or overlapped. The more separated these peaks are (less uncertainty), the larger the quality score is.

Three measures of uncertainty are currently implemented. The first one is the same as that defined in the `CNVtools` package, the second is the estimated probabilty of good classification (PGC), and the third is defined as the the proportion of individuals with a confidence score (described in [4]) bigger than 0.1.
To choose PGC method type

```
> CNVassoc::getQualityScore(CNV.1, type = "class")

--Probability of good classification: 0.9999963

> CNVassoc::getQualityScore(CNV.2, type = "class")

--Probability of good classification: 0.9096771
```

To choose the measure defined in the `CNVtools` package:

```
> CNVassoc::getQualityScore(CNV.1, type = "CNVtools")

--CNVtools Quality Score: 25.16849

> CNVassoc::getQualityScore(CNV.2, type = "CNVtools")

--CNVtools Quality Score: 3.057171
```

And to choose the third measure:

```
> CNVassoc::getQualityScore(CNV.1, type = "CANARY")

--Probability to have a 'CANARY confidence index' > 0.1 : 0

> CNVassoc::getQualityScore(CNV.2, type = "CANARY")

--Probability to have a 'CANARY confidence index' > 0.1 : 0.3024652
```

It is clear that in Gene 1 there is much less uncertainty, because the PGC is greater than 99%, the measure of `CNVtools` package is higher than 25 (CNVtools recommends a quality score of 4 or larger), or the "CANARY" measure is almost 0. This fact can also be seen in Figure 5 where the underlying distributions of signal intensity are very well separated. On the other hand, the PGC for Gene 2 is 91.3%, and the `CNVtools` package value is about 3 indicating that more uncertainty is present, and the "CANARY" type measure for Gene 2 tells that up to 30% of individuals have a poor confidence score. When cnv object has been created directly from probabilities (obtained from any other calling algorithm), only `type="CANARY"` method can be computed. In [5], it is suggested that, when proportion of individuals with confidence score > 0.1 is greater than 10%, this particular CNV should be removed from the analysis under a best-guess strategy in performing the association test.

2.5 Assessing associations between CNV and disease

The function `CNVassoc` carries out association analysis between CNV and disease. This function incorporates calling uncertainty by using a latent class model as described in [3]. The function can analyze both binary and quantitative traits. In the first case, a linear regression is performed, and, in the second, a logistic regression. The regression model can be selected by using the argument `case.control`. Nonetheless, the program automatically detects whether or not a quantitative trait is being analyzed so it need not be specified.

The function also allows the user to fit a model with additive or multiplicative effects of CNV. This can be set through the argument `model`. Possible values are "add" for an additive effect or "mul" for a multiplicative effect.

The function `CNVassoc` returns an object of class `CNVassoc`. This class of object has some properties in common with objects of class `glm`, such as `coef` or `summary` among others.

2.5.1 Modelling association

The effect of a given CNV on case/control status (`casco` variable) can be fitted by typing

```
> model1mul <- CNVassoc(casco ~ CNV.1, data = dataMLPA, model = "mul")
> model2mul <- CNVassoc(casco ~ CNV.2, data = dataMLPA, model = "mul")
```

By default, a short summary is printed (similar to `glm` objects)

```
> model1mul

Call:  CNVassoc(formula = casco ~ CNV.1, data = dataMLPA, model = "mul")

Coefficients:
         CNV0      CNV1      CNV2
CNVmult  0.0281709 0.5187566 1.0989109

Number of individuals: 651
Number of estimated parameters: 3
Deviance: 883.03

> model2mul

Call:  CNVassoc(formula = casco ~ CNV.2, data = dataMLPA, model = "mul")

Coefficients:
         CNV0        CNV1        CNV2
CNVmult   1.0520923   0.3122567  -0.0970782

Number of individuals: 651
Number of estimated parameters: 3
Deviance: 876.396
```

Note that the coefficients are a matrix with one row per variable and a column for each distinct copy number status. In this model, because there are no covariates and the CNV has a multiplicative effect, there is just one row (one intercept) and this is different among columns (copy number status).

By using the generic function `summary` we can obtain a more exhaustive output. In particular the odds ratio and its confidence intervals are printed as well as its p-value.

```
> summary(model1mul)

Call:
CNVassoc(formula = casco ~ CNV.1, data = dataMLPA, model = "mul")

Deviance: 883.0297
Number of parameters: 3
Number of individuals: 651

Coefficients:
         OR lower.lim upper.lim     SE   stat pvalue
CNV0 1.0000
CNV1 1.6333    1.1588    2.3020 0.1751 2.8017  0.005
CNV2 2.9175    1.1359    7.4937 0.4813 2.2247  0.026

(Dispersion parameter for  binomial  family taken to be  1 )


Covariance between coefficients:
     CNV0   CNV1   CNV2
CNV0 0.0094 0.0000 0.0000
CNV1        0.0213 0.0000
CNV2               0.2223

> summary(model2mul)

Call:
CNVassoc(formula = casco ~ CNV.2, data = dataMLPA, model = "mul")

Deviance: 876.396
Number of parameters: 3
Number of individuals: 651

Coefficients:
          OR lower.lim upper.lim      SE    stat   pvalue
CNV0  1.0000
CNV1  0.4772    0.2742    0.8304  0.2827 -2.6172    0.009
CNV2  0.3169    0.1834    0.5477  0.2791 -4.1169 3.84e-05

(Dispersion parameter for  binomial  family taken to be  1 )


Covariance between coefficients:
     CNV0    CNV1    CNV2
CNV0  0.0613  0.0000  0.0000
CNV1          0.0186 -0.0032
CNV2                  0.0166
```

By default, `CNVassoc` treats the response variable as a binary phenotype coded as 0/1. Since `CNVassoc` can handle other distributions such as Poisson or Weibull, the `family` argument must be

specified when the response is not distributed as a bernoulli. For instance, to deal with a normally distributed response variable, specify `family="gaussian"`

The following example presents the case of analyzing a quantitative normally distributed trait and adjusting the association by other covariates:

```
> mod <- CNVassoc(quanti ~ CNV.2 + cov, family = "gaussian", data = dataMLPA,
+     model = "add", emsteps = 10)
> mod

Call:  CNVassoc(formula = quanti ~ CNV.2 + cov, data = dataMLPA, model = "add",      family =

Coefficients:
           CNV0        CNV1        CNV2
intercept  -0.1403761  -0.1403761  -0.1403761
CNVadd     -0.0792367  -0.0792367  -0.0792367
cov         0.0241877   0.0241877   0.0241877

Number of individuals: 651
Number of estimated parameters: 4
Deviance: 1824.57
```

Notice that in this case, we use new argument called `emsteps`. This is necessary for computational reasons. Initially performing some preliminary steps using the EM algorithm makes it easier to maximize the likelihood function using the Newton-Raphson procedure. In general, it is enough to perform a few iterations (no more than 10). As usual, the model is then summarized by typing

```
> summary(mod)

Call:
CNVassoc(formula = quanti ~ CNV.2 + cov, data = dataMLPA, model = "add",
    family = "gaussian", emsteps = 10)

Deviance: 1824.573
Number of parameters: 4
Number of individuals: 651

Coefficients:
                beta lower.lim upper.lim       SE     stat pvalue
(Intercept) -0.14038  -0.90687   0.62612  0.39108 -0.35895  0.720
trend       -0.07924  -0.19714   0.03866  0.06015 -1.31722  0.188
cov          0.02419  -0.05068   0.09906  0.03820  0.63321  0.527

(Dispersion parameter estimation for  gaussian family is 0.9650261 )


Covariance between coefficients:
          intercept CNVadd  cov
intercept  0.1529   -0.0041 -0.0146
CNVadd               0.0036 -0.0001
cov                          0.0015
```

Remember that for quantitative traits we obtain mean differences instead of odds ratios.

2.5.2 Testing associations

In the previous analysis we obtained p values corresponding to the comparison between every copy number status versus the reference (zero copies). Nonetheless, we are normally interested in testing the overall effect of CNV on disease. We have implemented the Wald test and the likelihood ratio test (LRT) to perform such omnibus testing. Both are available through the function `CNVtest` which requires an object of class `CNVassoc` as the input. To specify the type of test, set the argument `type` to `"Wald"` or `"LRT"`, respectively. For Gene 1,

```
> CNVtest(model1mul, type = "Wald")

----CNV Wald test----
Chi= 11.55332 (df= 2 ) , pvalue= 0.003099052

> CNVtest(model1mul, type = "LRT")

----CNV Likelihood Ratio Test----
Chi= 12.12081 (df= 2 ) , pvalue= 0.002333458
```

and for Gene 2,

```
> CNVtest(model2mul, type = "Wald")

----CNV Wald test----
Chi= 17.32966 (df= 2 ) , pvalue= 0.0001725492

> CNVtest(model2mul, type = "LRT")

----CNV Likelihood Ratio Test----
Chi= 18.75453 (df= 2 ) , pvalue= 8.462633e-05
```

Other generic functions like `logLik`, `coef`, `summary` or `update` can be applied to an object of class `CNVassoc` to get more information.

For a multiplicative CNV effect model and for a binary traits, it is possible to change the reference category of copy number status. This can be done by using the argument `ref` when executing the `summary` function. For example, if we want to one copy as the reference category just type:

```
> coef(summary(model1mul, ref = 2))

            OR lower.lim upper.lim        SE      stat      pvalue
CNV1 1.0000000        NA        NA        NA        NA          NA
CNV0 0.6122677 0.4344016 0.8629612 0.1751053 -2.801661 0.005084028
CNV2 1.7863140 0.6790498 4.6990928 0.4934862  1.175624 0.239745087
```

The same kind of results can be obtained if we assume an additive effect of CNV on the trait. In this case we need to set the `model` argument to `"add"`

```
> model2add <- CNVassoc(casco ~ CNV.2, data = dataMLPA, model = "add")
> model2add
```

```
Call:  CNVassoc(formula = casco ~ CNV.2, data = dataMLPA, model = "add")

Coefficients:
           CNV0      CNV1      CNV2
intercept   0.932028   0.932028   0.932028
CNVadd     -0.537731  -0.537731  -0.537731

Number of individuals: 651
Number of estimated parameters: 2
Deviance: 877.061
```

Notice that under an additive CNV effect the structure of coefficients are different from the multiplicative CNV effect. Now there are two rows, one for intercept and the other one for the slope (change of risk in increasing by one copy). These two values remain constant for every column (copy number status).

```
> summary(model2add)

Call:
CNVassoc(formula = casco ~ CNV.2, data = dataMLPA, model = "add")

Deviance: 877.0606
Number of parameters: 2
Number of individuals: 651

Coefficients:
           OR lower.lim upper.lim      SE    stat   pvalue
trend  0.5841    0.4530    0.7530  0.1296 -4.1477 3.36e-05

(Dispersion parameter for  binomial  family taken to be  1 )


Covariance between coefficients:
          intercept CNVadd
intercept  0.0374   -0.0228
CNVadd               0.0168
```

Finally, one might be interested in testing the additive effect. To do this, one can compare both additive and multiplicative models. It is straightforward to see that the additive model is a particular case of the multiplicative one, and therefore the first is nested in the second one.

To compare two nested models we use the generic function `anova` (NOTE: it is only implemented for comparing two models, both fitted with the `CNVassoc` function).

```
> anova(model2mul, model2add)

--- Likelihood ratio test comparing 2 CNVassoc models:


Model 1 call:  CNVassoc(formula = casco ~ CNV.2, data = dataMLPA, model = "mul")

Model 2 call:  CNVassoc(formula = casco ~ CNV.2, data = dataMLPA, model = "add")
```

```
Chi= 0.6645798 (df= 1 )  p-value= 0.4149477

  Note: the 2 models must be nested, and this function doesn't check this!
```

The likelihood ratio test is performed. In this case the p-value is not significant, indicating that an additive CNV effect can be assumed. In any case, one should consider the power of this test before making conclusions.

2.6 Analysing other genetic models

In assessing copy number variant effect on a disease CNVassoc package can deal with additive or multiplicative (see more details in [3]). The first one ('additive') suppose an equal increase in logit of risk (for case-control studies) or in mean (for a quantitative traits) for example, while 'multiplicative' makes no assumptions on CNV effect.

In the particular case that CNV has 3 categories ('copy lose', 'normal' or 'copy gain'), it may be useful to assume CNV effect not being additive or multiplicative, but dominant or recessive. That is, to compare the effect of 'copy lose' vs. the other two if a dominant effect is assumed, or to compare 'copy gain' vs. the other two if a recessive effect is assumed.

It is possible to assesse such 'dominant' or 'recessive' effect using CNVassoc package functions. To do so, few simple steps have to be done before performing the associations analysis. Here, we illustrate the required instructions to perform the association analysis assuming a recessive or a dominant effect, taking MLPA example data already present in the CNVassoc package.

a) **Package and data loading:** First, CNVassoc package and MLPA data are loaded

```
> library(CNVassoc)
> data(dataMLPA)
```

b) **Inferring copy number status:** Then, CNV from Gene2 signal intensities is inferred. And its copy number probabilities matrix is stored in 'probs' object.

```
> CNV <- cnv(x = dataMLPA$Gene2, threshold.0 = 0.01, mix.method = "mixdist")
> CNV

Inferred copy number variant by a quantitative signal
   Method: function mix {package: mixdist}

-. Number of individuals: 651
-. Copies 0, 1, 2
-. Estimated means: 0, 0.2435, 0.4469
-. Estimated variances: 0, 0.0041, 0.0095
-. Estimated proportions: 0.1306, 0.4187, 0.4507
-. Goodness-of-fit test: p-value= 0.4887659


-. Note: number of classes has been selected using the best BIC

> probs <- attr(CNV, "probabilities")
```

c) **Updating CNV to dominant or recessive:** Once CNV is inferred, some previous modifications to CNV object have to be done: For assessing the recessive effect, first and second copy number status has to be joined. To do so, first and second columns of 'probs' are added. Also, copy number status labels must be modified from 'CNVrec' and 'CNVdom' objects. In both cases, they can be set to 0,1:

```
> probsrec <- cbind(rowSums(probs[, 1:2]), probs[, 3])
> CNVrec <- cnv(probsrec, num.copies = c(0, 1))
> CNVrec

-. Copy number variant
   Input data: called probabilities
-. Number of individuals: 651
-. Copies 0, 1
-. Estimated proportions: 0.5493, 0.4507
```

And for assessing the dominant effect, we proceed the same way but adding the second and third columns:

```
> probsdom <- cbind(probs[, 1], rowSums(probs[, 2:3]))
> CNVdom <- cnv(probsdom, num.copies = c(0, 1))
> CNVdom

-. Copy number variant
   Input data: called probabilities
-. Number of individuals: 651
-. Copies 0, 1
-. Estimated proportions: 0.1306, 0.8694
```

d) **Performing association test:** Finally, association analysis is performed as usual, specifying a 'multiplicative' effect in the 'model' argument (note that an 'additive' effect could be set and the same results would be obtained). In this example, the Odds Ratio of category labelled as '1' vs '0' is displayed. When assessing the dominant model effect, '1' will contain 'copy-gain' and 'normal' categories, while '0' will contain 'copy-lose'. On the other hand, when assessing the recessive model effect, '1' will contain 'copy-gain', and '0' will contain 'normal' and 'copy-lose' categories.

The results of the association test are:

- for recessive

```
> summary(CNVassoc(casco ~ CNVrec, data = dataMLPA))
Call:
CNVassoc(formula = casco ~ CNVrec, data = dataMLPA)

Deviance: 883.644
Number of parameters: 2
Number of individuals: 651

Coefficients:
         OR lower.lim upper.lim     SE    stat pvalue
CNV0  1.0000
CNV1  0.5309    0.3672    0.7677  0.1882 -3.3650  0.001
```

```
(Dispersion parameter for  binomial  family taken to be  1 )


Covariance between coefficients:
     CNV0    CNV1
CNV0  0.0141 -0.0024
CNV1          0.0165
```

- and for dominant

```
> summary(CNVassoc(casco ~ CNVdom, data = dataMLPA))
Call:
CNVassoc(formula = casco ~ CNVdom, data = dataMLPA)

Deviance: 880.4668
Number of parameters: 2
Number of individuals: 651

Coefficients:
          OR lower.lim upper.lim      SE    stat   pvalue
CNV0  1.0000
CNV1  0.3856    0.2309    0.6438  0.2616 -3.6438 0.000269

(Dispersion parameter for  binomial  family taken to be  1 )


Covariance between coefficients:
     CNV0   CNV1
CNV0 0.0613 0.0000
CNV1        0.0071
```

3 CNV from aCGH

The analysis of aCGH data requires taking additional steps into account, due to the dependency across probes and the fact that CNVs are not measured with a unique probe. Table 1 shows four steps we recommend for the analysis of this kind of data. First, posterior probabilities should be obtained with an algorithm that considers probe correlation. We use, in particular, the `CGHcall` `R` program which includes a mixture model to infer CNV status [9]. Second, we build blocks/regions of consecutive clones with similar signatures. To perform this step the `CGHregions` `R` library was used [10]. Third, the association between the CNV status of blocks and the trait is assessed by incorporating the uncertainty probabilities in `CNVassoc` function. And fourth, corrections for multiple comparisons must be performed. We use the Benjamini-Hochberg(BH) correction [2]. This is a heuristic method that is robust against positive dependence and increasingly conservative as correlation increases.

To illustrate, we apply these steps to the breast cancer data studied by Neve et al. [6]. The data consists of CGH arrays of 1MB resolution and is available from Bioconductor `http://www.bioconductor.org/`. The authors chose the 50 samples that could be matched to the name tokens of caArrayDB data (June 9th 2007). In this example the association between strogen receptor positivity (dichotomous variable; 0: negative, 1: positive) and CNVs was tested. The original data set contained 2621 probes which were reduced to 459 blocks after the application of `CGHcall` and `CGHregions` functions as we illustrate bellow.

Table 1: Steps to assess association between CNVs and traits for aCGH

**Step 1**.	Use any aCGH calling procedure that provides posterior probabilities (uncertainty) (`CGHcall`)
**Step 2**.	Build blocks/regions of consecutive probes with similar signatures (`CGHregions`)
**Step 3**.	Use the signature that occurs most in a block to perform association(`multiCNVassoc`)
**Step 4**.	Correct for multiple testing considering dependency among signatures (`getPvalBH`)

The data is saved in an object called `NeveData`. This object is a list with two components. The first component corresponds to a dataframe containing 2621 rows and 54 columns with aCGH data (4 columns for the annotation and 50 log2ratio intensities). The second component is a vector with the phenotype analyzed (strogen receptor posistivity). The data can be loaded as usual

```
> data(NeveData)
> intensities <- NeveData$data
> pheno <- NeveData$pheno
```

The calling can be performed using `CGHcall` package by using the following instructions:

```
\dontrun{
#######################################################
### chunk number 1: Class of aCGH data
#######################################################
library(CGHcall)
Neve <- cghRaw(intensities)

#######################################################
### chunk number 2: Preprocessing
#######################################################
cghdata <- preprocess(Neve, maxmiss=30, nchrom=22)

#######################################################
### chunk number 3: Normalization
#######################################################
norm.cghdata <- normalize(cghdata, method="median", smoothOutliers=TRUE)

#######################################################
### chunk number 4: Segmentation
#######################################################
seg.cghdata <- segmentData(norm.cghdata, method="DNAcopy")

#######################################################
### chunk number 5: Calling
#######################################################
NeveCalled <- CGHcall(seg.cghdata)
}
```

This process takes about 20 minutes, but to avoid wasting your time, we have saved the final object of class `cghCall` that can be loaded as

```
> data(NeveCalled)
```

We can then obtain the posterior probabilities. `CGHcall` function does not estimates the underlying number of copies for each segment but assigns the underlying status: loss, normal or gain. For each segment and for each individual we obtain three posterior probabilities corresponding to each of these three statuses. This is done by executing

```
> probs <- getProbs(NeveCalled)
```

This is a dataframe that looks like this:

```
> probs[1:5, 1:7]
```

```
                 Clone Chromo BPstart   BPend X600MPE X600MPE.1 X600MPE.2
RP11-82D16 RP11-82D16      1 2008651 2008651   0.022     0.932     0.046
RP11-62M23 RP11-62M23      1 3367844 3367844   0.022     0.932     0.046
RP11-111O5 RP11-111O5      1 4261844 4261844   0.022     0.932     0.046
RMC01P070   RMC01P070      1 5918606 5918606   0.022     0.932     0.046
RP11-51B4   RP11-51B4      1 6068980 6068980   0.022     0.932     0.046
```

This table can be read as following. The probability that the individual `X600MOE` is normal for the signature `RP11-82D16` is 0.932, while the probability of having a gain is 0.046 and 0.022 of having a loss.

In order to determine the regions that are recurrent or common among samples, we use the `CGHregions` function that takes an object of class `cghCall` (e.g. object `NeveCalled` in our case). This algorithm reduces the initial table to a smaller matrix that contains regions rather than individual probes. The regions consist of consequtive clones with similar signatures [10]. This can be done by executing

```
\dontrun{
library(CGHregions)
NeveRegions <- CGHregions(NeveCalled)
}
```

This process takes about 3 minutes. We have stored the result in the object `NeveRegions` that can be loaded as usual

```
> data(NeveRegions)
```

Now we have to get the posterior probabilities for each block/region. This can be done by typing

```
> probsRegions <- getProbsRegions(probs, NeveRegions, intensities)
```

Finally, the association analysis between each region and the strogen receptor positivity can be analyzed by using the `multiCNVassoc` function. This function repeatedly calls `CNVassoc` returning the p-value of association for each block/region

```
> pvals <- multiCNVassoc(probsRegions, formula = "pheno~CNV", model = "mult",
+     num.copies = 0:2, cnv.tol = 0.01)
```

Notice that the arguments of `multiCNVassoc` function are the same as those of `CNVassoc`. In this example, we have set the argument `num.copies` equal to 0, 1, and 2 that corresponds to `loss, normal, gain` status used in the `CGHcall` function.

Multiple comparisons can be addressed by using the Benjamini & Hochberg approach [2]. The function `getPvalBH` produces the corrected p-values

```
> pvalsBH <- getPvalBH(pvals)
> head(pvalsBH)
```

```
  region         pval     pval.BH
1    319 2.891862e-06 0.001324473
2    318 1.633799e-05 0.002494267
3    320 1.576279e-05 0.002494267
4    316 8.998845e-05 0.010303677
5      9 2.865773e-04 0.011217002
6    298 2.027325e-04 0.011217002
```

Table 6 in [3] can be obtained by typing

```
> cumsum(table(cut(pvalsBH[, 2], c(-Inf, 1e-05, 1e-04, 0.001, 0.01,
+     0.05))))
```

```
 (-Inf,1e-05] (1e-05,0.0001] (0.0001,0.001]   (0.001,0.01]    (0.01,0.05]
            1              4             27             64            117
```

4 Illumina data

In this section an example set of data from ILLUMINA will be analyzed. This data is included in the `CNVassoc` package, and is the same one as analyzed in the `CNVtools` package vignette [8]. The goal of this section will be to compare the results yielded by `CNVtools` in fitting the association model with those obtained with the `CNVassoc` function.

A first look at the data

```
> data(A112)
```

```
> head(A112)
```

```
          subject cohort        SNP0       SNP1       SNP2       SNP3
1 WTCCC01-11474A1    58C -0.12647400 -0.1214220 -0.1423570  0.0449446
2 WTCCC01-11474A2    58C -0.21574200  0.0265778 -0.0964269  0.0617480
3 WTCCC01-11474A3    58C -0.00150499  0.0820076 -0.2853430  0.1589580
4 WTCCC01-11474A4    58C -0.05538290 -0.1691450 -0.0592800  0.0264289
5 WTCCC01-11474A5    58C -0.12926900  0.2014540 -0.8474870 -0.2647420
6 WTCCC01-11474A6    58C -0.06209860  0.1826130  0.1245160 -0.1731720
        SNP4       SNP5       SNP6        SNP7        SNP8       SNP9
1  0.0259435  0.1351870  0.0746991  0.40581000 -0.18601600  0.0990579
2  0.1521360 -0.0445652 -0.3751110 -0.39122600  0.10114500  0.1816270
3  0.0320422  0.1823220  0.0699921  0.29014900  0.00885492 -0.0387201
4 -0.0208353 -0.2740840  0.0310302  0.20566300  0.12842100 -0.2219500
5 -0.0502723 -0.2150250 -0.2254730  0.00162372  0.08069250  0.0562238
6 -0.0870918 -0.0902743 -0.0634414 -0.80391700  0.37845800 -0.1880560
       SNP10      SNP11      SNP12       SNP13     SNP14      SNP15      SNP16
1 -0.1969750  0.0448241 -0.0193997  0.13117800 -0.163383  0.1545760  0.0253607
2  0.0688791 -0.1166620  0.0217019 -0.05719720 -0.138044 -0.0554405 -0.0536655
3  0.1131100  0.0609800  0.2402140  0.23635400 -0.111235  0.5082330  0.0272966
4 -0.2299260  0.0198905 -0.3210060  0.14955900 -0.534339 -0.7596830 -0.1940050
5 -0.0636589 -0.0433160 -0.5579070  0.13913000 -0.778225 -0.9224910  0.0343805
6 -0.1368910 -0.0779523  0.1212290  0.00857489  0.179257 -0.0675581 -0.1812210
       SNP17      SNP18     SNP19      SNP20      SNP21      SNP22      SNP23
1 -0.0560689 -0.0751385 -0.485160 -0.0288187 -0.1945410 -0.0456346  0.0929479
2  0.1212250  0.1018410 -0.200404 -0.1797650 -0.0456029  0.2835270 -0.0813351
3 -0.1532160 -0.1135340  0.183407 -0.0960403  0.1230410 -0.1076840 -0.0180287
4 -0.1035420  0.1661650 -0.318173 -0.7149550 -0.7436040 -0.2483910 -0.2552810
5 -0.0130905  0.1538550 -0.589194 -0.4773230 -0.6345150  0.1788480 -0.4428020
```

```
6 -0.1676740  0.3261350 -0.199970  0.0908316  0.1268390  0.1787620  0.1138070
      SNP24     SNP25      SNP26       SNP27      SNP28        SNP29      SNP30
1 -0.222375 -0.368043 -0.1448800 -0.00706918  0.0356588 -0.346104000 -0.1318280
2 -0.143908 -0.105819 -0.2330800 -0.07807670  0.0980952 -0.152811000  0.0728393
3  0.401502  0.240364 -0.1334340 -0.00942116 -0.0514102 -0.254315000  0.0708932
4 -0.106774  0.203908  0.3008000 -0.24017000  0.1681400  0.298436000  0.1303020
5 -0.124996 -0.191220 -0.1863940 -0.08408520 -0.2589270 -0.000203031 -0.0516899
6 -0.228779 -0.409863  0.0208064  0.01472170  0.2187790 -0.384239000 -0.0265277
       SNP31      SNP32
1  0.0140277  0.0583939
2 -0.2176910  0.0172098
3 -0.1251580 -0.1050300
4 -0.0139433  0.0432413
5  0.0381275 -0.0992932
6 -0.2410430 -0.0577618
```

In this case, intstead of having just one signal, a considerable number of them define a single gene. In CNVtools vignette [8] these are all summarized using principal components analysis, and the first component is taken in order to obtain one signal value per individual. The following steps to obtain peak intensities are the same as in [8].

To begin, load `CNVtools` package, since some function from it will be used to execute some previous steps in order to mimic the analysis performed in [8]:

```
> library(CNVtools)
```

4.1 Preparing signal data

The raw signal from all probes of the data is subtracted typing

```
> raw.signal <- as.matrix(A112[, -c(1, 2)])
> dimnames(raw.signal)[[1]] <- A112$subject
```

Then, the unidimensional data is summarized using principal component techinque from raw signal data

```
> pca.signal <- apply.pca(raw.signal)
```

In the article on `CNVtools` [1] it is suggested not to use this summarized intensity, `pca.signal`. Instead, the probability of occurrence of each of the 3 copy number status values (loss, normal and gain) is estimated after fitting a normal-mixture model to `pca.signal` using the function `CNVtest.binary` from CNVtools package.

```
> ncomp <- 3
> batches <- factor(A112$cohort)
> sample <- factor(A112$subject)
> fit.pca <- CNVtest.binary(signal = pca.signal, sample = sample,
+     batch = batches, ncomp = ncomp, n.H0 = 3, n.H1 = 0, model.var = "~ strata(cn)")
```

and after this, a linear discriminant analysis on raw signal data and these probabilities is performed

```
> pca.posterior <- as.matrix((fit.pca$posterior.H0)[, paste("P",
+     seq(1:ncomp), sep = "")])
> dimnames(pca.posterior)[[1]] <- (fit.pca$posterior.H0)$subject
> ldf.signal <- apply.ldf(raw.signal, pca.posterior)
```

4.2 Inferring copy number status considering batch effect

Once all signal probe intensities from the same gene have been summarized (`ldf.signal`), regardless of the techinque used, a normal mixture model is fitted using function `cnv` as already explained in Section 2. A possible batch effect in inferring copy number status has been considered, as mentioned

in [1]. Therefore, and in order to better mimic the example as presented in CNVtools vignette [8], copy number status is inferred taking into account the batches, simply by incorporating an argument to function `cnv`, called `batches`:

```
> CNV <- cnv(ldf.signal, batches = batches, num.class = 3, mix = "mclust")
> CNV

Inferred copy number variant by a quantitative signal
   Method: function Mclust {package: mclust}

-. Number of individuals: 2593
-. Copies 1, 2, 3
-. Estimated means:
      CNV 1   CNV 2  CNV 3
58C -1.9703 -0.2361 0.7752
NBS -2.1398 -0.1708 0.9074
-. Estimated variances:
     CNV 1  CNV 2  CNV 3
58C 0.0941 0.0941 0.0941
NBS 0.0847 0.0847 0.0847
-. Estimated proportions: 0.1524, 0.4973, 0.3503
```

In this case, the method "mclust" has been used in order to make the mixture model converge. Thus, a normal mixture is fitted separately per batch, and copy number status probability is updated pooling the copy number frequency among all batches. Notice that although specific means and variances are estimated per batch, only one pooled set of copy number frequencies is produced.

Also note that `plot` behaves slightly differently for CNV estimated taking into account the batch effect, drawing specific density curves and mean lines for each batch (see figure 7)

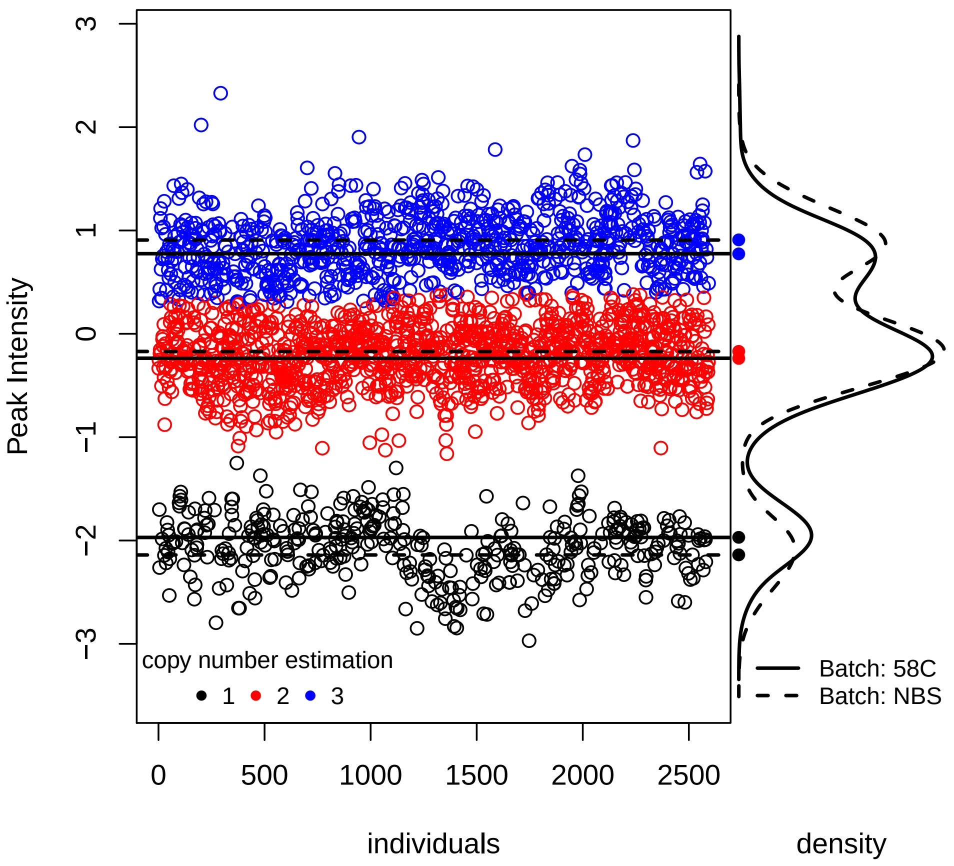

Figure 7: Signal distribution and inferred number of copies by batch

4.3 Association model: comparison with results from `CNVtools`

Now, the same batch variable will be the response as in [8], and an association model considering and additive effect test will be fitted. Since there are only 2 batches, a logistic regression will be performed. To compute the Likelihood Ratio Test on CNV:

```
> trait <- ifelse(A112$cohort == "58C", 0, 1)
> fit <- CNVassoc(trait ~ CNV, model = "add")
> CNVtest(fit, "LRT")

----CNV Likelihood Ratio Test----
Chi= 1.812608 (df= 1 ) , pvalue= 0.1781957
```

This results in a $\chi^2 = 1.81$ which does not differ greatly from the one given in CNVtools vignette [8] (1.55), neither being statistically significant.

And if a multiplicative model is assumed,

```
> fit <- CNVassoc(trait ~ CNV)
> CNVtest(fit, "LRT")

----CNV Likelihood Ratio Test----
Chi= 2.860054 (df= 2 ) , pvalue= 0.2393024
```

a χ^2 of 2.86 is obtained, similar to that in CNVtools-vignette [8] (3.11). Again, neither is statistically significant.

4.3.1 Power and computation time of CNVassoc and CNVtools

We simulated, under the same conditions as used by [1], a range of scenarios with different sample sizes, probe signal intensity distributions, etc., in order to explore the behavior of both methods when the copy number signals are not clearly separated. We observe that both methods performed well although CNVassoc outperforms CNVtools in the case of having a moderate number of individuals (e.g. 500) , see figures 8 and 9. However, we encounter and important problem of practical relevance related to convergence. CNVtools frequently fails to converge with moderate sample sizes: with 500 cases and 500 controls and $Q = 3$ [1], CNVtools failed to converge in more than 75% of the simulations and this failure rate reached 86% when $Q = 2.5$. Even with much larger sample sizes (2,000 cases and 2,000 controls) CNVtools failed to converge in 38% of the simulations when $Q = 2.5$. In constrast, CNVassoc converged in all scenarios with large sample size (2,000 and 2,000 controls) and with moderate sample sizes (500 cases and 500 controls) CNVassoc did not fail under low/moderate uncertainty $Q \geq 3.5$ and failed but much less than CNVtools when $Q \leq 3$, see table 2. Thus, for many studies being analyzed currently, CNVtools simply cannot provide a solution. When a solution is reached, the high rate of failure to converge raises questions about possible biases and imprecision of the results and, in any case, the solution is unlikely to be powerful enough to detect an association between copy number and phenotype.

We have also observed a marked difference in the speed of each procedure: when analyzing 10,000 CNVs in 2,000 cases and 2,000 controls, and with a $Q = 4$, CNVtools took 1 day and 17 hours to complete the analysis, whereas CNVassoc took just 90 minutes; with $Q = 3$, CNVtools took 6 days and 16 hours, but CNVassoc took only 2 hours.

[1] Q is the measure of uncertainty in inferring copy number status defined by CNVtools package (obtained by specifiyng the argument `type="CNVtools"` in `getQualityScore` function)

	$N = 2000$		$N = 500$	
Q	CNVassoc	CNVtools	CNVassoc	CNVtools
6.0	0	0	0	15
5.5	0	0	0	20
5.0	0	0	0	65
4.5	0	0	0	92
4.2	0	0	0	187
4.0	0	0	0	246
3.7	0	0	0	294
3.5	0	1	0	299
3.2	0	13	212	389
3.0	0	65	331	400

Table 2: Number of failed convergence simulations out of 500 using CNVassoc and CNVtools according to inferring copy number uncertainty Q and number of cases N.

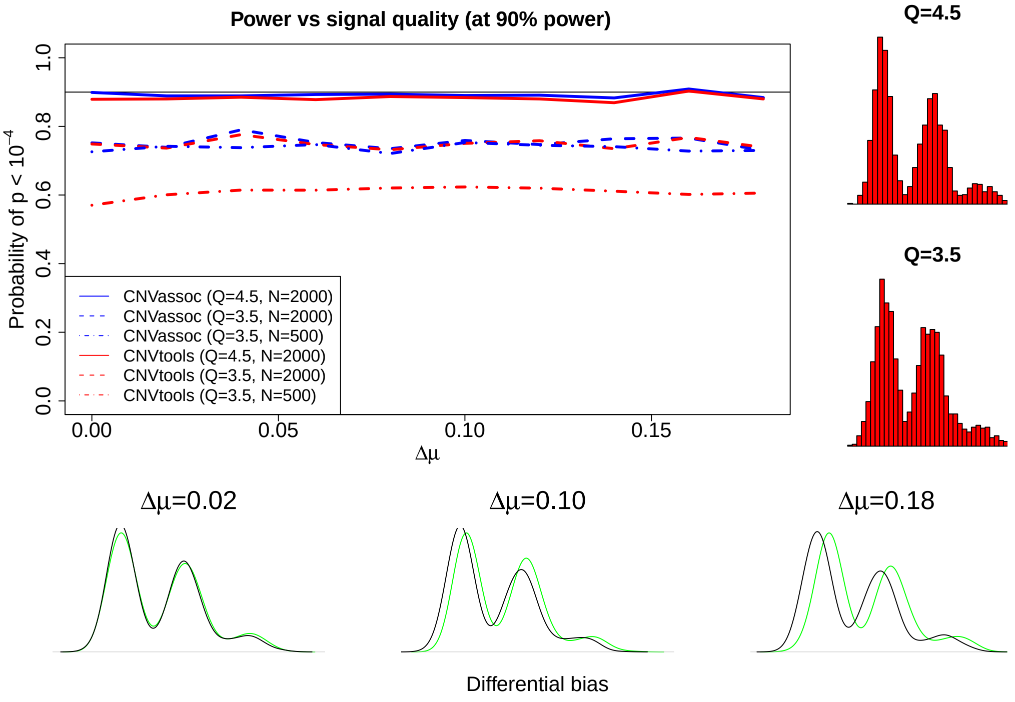

Figure 8: Power achieved by CNVassoc and CNVtools, depending on sample size, inferring copy number uncertainty (Q), degree of differencial bias ($\Delta\mu$) and sample size (N) under an scenario where the power to decect associated CNV is 90% if no inferring copy number uncertainty was present.

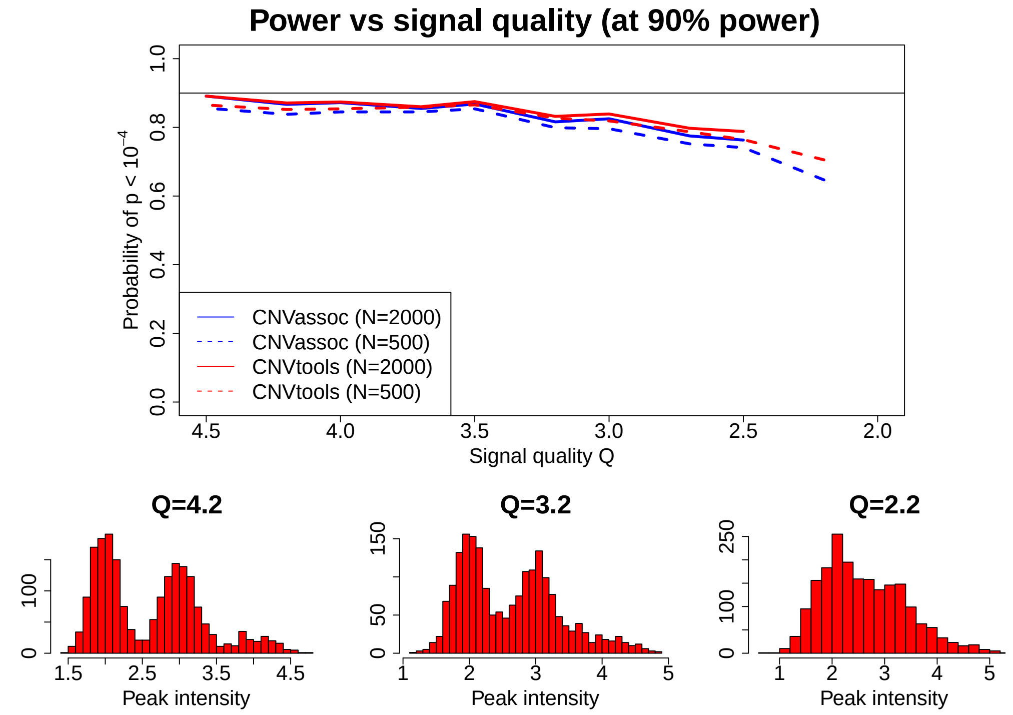

Figure 9: Power achieved by CNVassoc and CNVtools, with different values of copy number uncertainty (Q) and sample size (N) under an scenario where the power to decect associated CNV is 90% if no inferring copy number uncertainty was present.

5 Imputed data (SNPTEST format)

In this section we will show how `CNVassoc` can also be used to analyse SNP data when the SNPs have been imputed or genotyped with some degree of error. Notice that the same procedure can be applied to analyze data from Birdsuite/Canary software (developed by Broad Institute and available on http://www.broadinstitute.org/). An example from SNPTEST software (available on http://www.stats.ox.ac.uk/ [illegible] chini/software/gwas/snptest.html) has been incorporated in the `CNVassoc` package, but in the same format as used by IMPUTE software (downloable from SNPTEST website). IMPUTE is a program to infer a set non observed SNPs from other that have been genotyped, using linkage desequilibrium and other information, usually from the HapMap project (http://snp.cshl.org/). The data of the following example can be downloaded freely from the SNPTEST software website, and consists of a set of 500 cases and 500 controls, and 100 SNPs. For all of the SNPs the probabilities of each genotype is given, not the genotype itself, simulating having been obtained from IMPUTE. The names of the SNPs have been masked, as also the name of the disease.

Let's load the data. There are 2 data frames, one for cases and the other for controls

```
> data(SNPTEST)
> dim(cases)
```

```
[1]  100 1505
```

```
> dim(controls)
```

```
[1]  100 1505
```

```
> cases[1:10, 1:11]
```

```
   V1 V2    V3 V4 V5           V6           V7           V8          V9
1   1  1  1000  A  T 0.9959626125 0.0023620260 0.0016753615 0.992634932
2   2  2  2000  A  T 0.0765213302 0.0073893102 0.9160893596 0.027811741
3   3  3  3000  A  T 0.0050670931 0.0020722897 0.9928606172 0.009646064
4   4  4  4000  A  T 0.9920997158 0.0003108851 0.0075893991 0.012288000
5   5  5  5000  A  T 0.0048796013 0.0283927739 0.9667276249 0.990459821
6   6  6  6000  A  T 0.0029449045 0.9965970143 0.0004580812 0.993531065
7   7  7  7000  A  T 0.9844537961 0.0147126387 0.0008335652 0.003635098
8   8  8  8000  A  T 0.0002854996 0.0019421881 0.9977723123 0.005000345
9   9  9  9000  A  T 0.0052202003 0.0037747406 0.9910050592 0.003845385
10 10 10 10000  A  T 0.0145463505 0.9603995477 0.0250541018 0.010122825
            V10          V11
1  0.0003516265 7.013442e-03
2  0.0086429180 9.635453e-01
3  0.0026860830 9.876679e-01
4  0.9815783730 6.133627e-03
5  0.0092745162 2.656632e-04
6  0.0023760942 4.092840e-03
7  0.9945822710 1.782631e-03
8  0.0024962428 9.925034e-01
9  0.0011333510 9.950213e-01
10 0.9898094554 6.771937e-05
```

```
> controls[1:10, 1:11]
```

```
   V1 V2    V3 V4 V5           V6          V7          V8           V9
1   1  1  1000  A  T 9.822425e-01 0.003358295 0.014399242 0.9910275077
2   2  2  2000  A  T 1.333922e-02 0.969099360 0.017561421 0.0070884674
3   3  3  3000  A  T 3.989599e-03 0.004256366 0.991754036 0.0014208265
4   4  4  4000  A  T 3.406932e-03 0.007333515 0.989259553 0.0006075389
5   5  5  5000  A  T 9.881081e-01 0.010474830 0.001417104 0.9828012172
6   6  6  6000  A  T 3.595319e-03 0.990430376 0.005974305 0.0003284885
```

```
7   7  7  7000  A  T 6.072451e-05 0.997494894 0.002444382 0.0034642921
8   8  8  8000  A  T 6.322546e-03 0.006265613 0.987411841 0.0016109147
9   9  9  9000  A  T 3.073608e-04 0.007901964 0.991790675 0.0160832317
10 10 10 10000  A  T 9.748969e-03 0.978622828 0.011628203 0.0076508106
          V10         V11
1  0.001110983 0.007861509
2  0.028424366 0.964487167
3  0.984644304 0.013934870
4  0.997842168 0.001550293
5  0.011371321 0.005827462
6  0.995963534 0.003707978
7  0.989251393 0.007284314
8  0.006935266 0.991453820
9  0.981741626 0.002175142
10 0.973590298 0.018758891
```

The structure of the data is as follows:

- every row is a SNP
- the first 3 columns are the SNP identification codes,
- the 4th and 5th are the alleles.
- columns 6 through to the end provide the probabilities of each genotype, each group of 3 columns corresponds to one individual.

For example, the first individual in the data set of cases has probabilities of 0.996, 0.0024 and 0.0017 of having the genotypes for the first SNP of AA, AT and TT respectively. And the second individual has a probabilities of 0.0278, 0.0086 and 0.9635 of having the genotypes for the second SNP of AA, AT and TT respectively.

Of course, cases and controls must have the same number of rows, because the i-th row of cases and the i-th row of controls correspond to the same SNP.

First in order to use `CNVassoc` certain preliminary data management steps are needed. The goal is to have one matrix of probabilities with 3 columns corresponding to the 3 genotypes and 1000 individuals (500 cases plus 500 controls), for each of the 100 SNPs.

```
> nSNP <- nrow(cases)
> probs <- lapply(1:nSNP, function(i) {
+     snpi.cases <- matrix(as.double(cases[i, 6:ncol(cases)]),
+         ncol = 3, byrow = TRUE)
+     snpi.controls <- matrix(as.double(controls[i, 6:ncol(controls)]),
+         ncol = 3, byrow = TRUE)
+     return(rbind(snpi.cases, snpi.controls))
+ })
```

Now `probs` is a list of 100 components, each one containing the probability matrix of each SNP, and the first 500 rows of each matrix refers to the cases and the rest to the controls.

In this point, we can use `multiCNVassoc` as shown in section 3, to perform an association test of each SNP with case control status. But first, a casecontrol variable must be defined, which, in this example, will be a simple vector of 500 ones and 500 zeros.

```
> casecon <- rep(1:0, c(500, 500))
```

Now, we have the data ready to fit a model. For example, to compute the association p-value between every SNP and case control status assuming an additive effect:

```
> pvals <- multiCNVassoc(probs, formula = "casecon~CNV", model = "add",
+     num.copies = 0:2, cnv.tol = 0.001)
```

And, as in section 3, it is necessary to correct for multiple tests:

```
> pvalsBH <- getPvalBH(pvals)
> head(pvalsBH)

  region       pval   pval.BH
1      1 0.29083371 0.8400958
2      3 0.13235295 0.8400958
3      5 0.08296301 0.8400958
4      6 0.18826664 0.8400958
5      7 0.24967318 0.8400958
6      9 0.30321197 0.8400958
```

A frecuency tabulation of how many SNP achieve different levels of significance is obtained by:

```
> table(cut(pvalsBH[, 2], c(-Inf, 0.001, 0.01, 0.05, 0.1, Inf)))

(-Inf,0.001] (0.001,0.01]  (0.01,0.05]   (0.05,0.1]   (0.1, Inf] 
           0            0            2            7           91 
```

From these results, no SNP appears to be associated with case control status.

6 Other phenotype distributions

The examples of the previous section dealt with continuous normally distributed phenotypes, and binary traits. However, there are situations where we may be interested in associating CNV with a phenotype that is not normally distributed, or which is not a binary trait.

6.1 Poisson distributed phenotype

One example of a phenotype that doesn't fit with previous examples is a counting process, that could be the number of times that a patient replapses from a specific cancer. This could be modelled with a Poisson distribution.

`CNVassoc` incorporates the possibility to fit a Poisson distribution by specifying `family`="poisson". Also, `CNVassoc` has a function to simulate CNV data and Poisson phenotype. Therefore, in this section simulated data from this function will be analysed.

Data for 4000 individuals has been simulated under the following scenario:

- CNV copy number of 0, 1 and 2 with probabilities of 0.25, 0.5 and 0.25 respectively,
- CNV intensity signal means of 0, 1 and 2 for 0, 1 and 2 copies respectively,
- CNV intensity signal standard deviation of 0.4 for each copy,
- an additive effect with a risk ratio of 1.7 for each increment in copy number status,
- incidence of 0.12 of relapsing among individuals with zero copies (which means a probability of 0.6737 of having at least one relapse).

```
> set.seed(123456)
> rr <- 1.7
> incid0 <- 0.12
> lambda <- c(incid0, incid0 * rr, incid0 * rr^2)
> dsim <- simCNVdataPois(n = 4000, mu.surrog = 0:2, sd.surrog = rep(0.4,
+     3), w = c(0.25, 0.5, 0.25), lambda = lambda)
> head(dsim)

     resp cnv     surrog
446     0   1  0.1626554
2214    0   2  1.1287803
3535    1   3  1.4992945
3579    1   3  1.9024086
678     0   1 -0.2533025
2813    2   2  0.4879491
```

The result is a data frame with 3 variables, and as many rows as individuals. The description of these variables is:

- `resp`: response, distributed as a Poisson given the copy number status,
- `cnv`: the real copy number status, which, in practice, will be unknown and not considered in testing the association,
- `surrog`: the CNV intensity signal.

First an object of class `cnv` is obtained fitting a normal mixture to the intensity signal, as in section ... Note that to make the normal mixture converge "mclust" method is specified:

```
> CNV <- cnv(dsim$surrog, mix = "mclust")
> CNV

Inferred copy number variant by a quantitative signal
   Method: function Mclust {package: mclust}

-. Number of individuals: 4000
-. Copies 1, 2, 3
-. Estimated means: 0.0141, 0.9774, 1.9636
-. Estimated variances: 0.1631, 0.1631, 0.1631
-. Estimated proportions: 0.2479, 0.4804, 0.2717


-. Note: number of classes has been selected using the best BIC
```

Note that 3 copy number status have been inferred by BIC criteria. By default 1, 2 and 3 copies are assigned. To change the copy number status to 0, 1 and 2 copies, just change the `num.copies` attribute properly:

```
> attr(CNV, "num.copies") <- 0:2
> CNV
```

```
Inferred copy number variant by a quantitative signal
   Method: function Mclust {package: mclust}

-. Number of individuals: 4000
-. Copies 0, 1, 2
-. Estimated means: 0.0141, 0.9774, 1.9636
-. Estimated variances: 0.1631, 0.1631, 0.1631
-. Estimated proportions: 0.2479, 0.4804, 0.2717


-. Note: number of classes has been selected using the best BIC
```

Then, an association model with CNV and the phenotype assuming an additive effect is performed as usual, but specifying `family="poisson"` in the call to function `CNVassoc`:

```
> fit <- CNVassoc(resp ~ CNV, data = dsim, family = "poisson",
+     model = "add")
> coef(summary(fit))
```

```
            RR lower.lim upper.lim         SE     stat pvalue
trend 1.613005  1.450285  1.793982 0.05425561 8.811971      0
```

The same generic functions are appliable as for normal and binary traits. Note that, now, `summary` prints "RR" instead of "OR".

We can compare this to the "gold standard" model, where the phenotype is regressed to the true copy number status:

```
> fit.gold <- glm(resp ~ cnv, data = dsim, family = "poisson")
> table.gold <- c(exp(c(coef(fit.gold)[2], confint(fit.gold)[2,
+     ])), coef(summary(fit.gold))[2, 4])
> names(table.gold) <- c("RR", "lower", "upper", "p-value")
> table.gold
```

```
          RR        lower        upper      p-value
1.701183e+00 1.547603e+00 1.871468e+00 5.752637e-28
```

The confidence interval of the estimate contains the true relative risk, and the "gold standard" model gives similar results as the one fitted using `CNVassoc` function (latent class model).

Because the data has been simulated from a fixed scenario, we may be interested in comparing with an estimation made under a naive strategy, i.e. compared to fitting a standard log-linear Poisson model assigning the most probable copy number to each individual (best guess approach):

```
> fit.naive <- glm(resp ~ CNV, data = dsim, family = "poisson")
> table.naive <- c(exp(c(coef(fit.naive)[2], confint(fit.naive)[2,
+     ])), coef(summary(fit.naive))[2, 4])
> names(table.naive) <- c("RR", "lower", "upper", "p-value")
> table.naive
```

```
          RR        lower        upper      p-value
1.555179e+00 1.415058e+00 1.710412e+00 6.646768e-20
```

To sum up, table 3 gives the relative risk estimated under different models (gold standard, latent class and naive):

	RR	lower	upper
Gold	1.70	1.55	1.87
LC	1.61	1.45	1.79
Naive	1.56	1.42	1.71

Table 3: Comparison of RR estimated by the gold standard model, a latent class model (LC) and naive approach

6.2 Weibull distributed phenotype

Similarly to a Poisson distributed phenotype, we may be interested in fitting data that comes from a followed cohort, where we want to estimate associations of time to death or onset of a particular disease with copy number variant. Probably some individuals will be censored, i.e. at the end of follow-up they are alive or free of disease. As for classical survival analysis is important to take into account these censored individuals and not to remove them from the analysis.

Function `CNVassoc` can handle this situation, simply by specifying `family`=”weibull” rather than poisson or gaussian. In considering censoring status, function `Surv` must be invoked in the left hand term of the formula argument (as for `coxph` function for example).

In this subsection we illustrate how to fit a model with time to event, possibly censored, by fitting simulated data, in a similar manner to the previous subsection (Poisson distributed phenotype), and using function `simCNVdataWeibull` implemented in the CNVassoc package.

The following scenario has been simulated for 5000 individuals:

- CNV copy number of 0, 1 and 2 with probabilities of 0.25, 0.5 and 0.25 respectively,
- CNV intensity signal means of 0, 1 and 2 for 0, 1 and 2 copies respectively,
- CNV intensity signal standard deviation of 0.4 for each copy,
- an additive effect with a hazard ratio of 1.5 for each increment of copy number status
- shape parameter of the weibull distribution equal to one,
- disease incidence equal to 0.05 (per person-year) among the population with zero copies.
- proportion of non-censored individuals (who suffered the disease during the study) of 10%.

```
> set.seed(123456)
> n <- 5000
> w <- c(0.25, 0.5, 0.25)
> mu.surrog <- 0:2
> sd.surrog <- rep(0.4, 3)
> hr <- 1.5
> incid0 <- 0.05
> lambda <- c(incid0, incid0 * hr, incid0 * hr^2)
> shape <- 1
> scale <- lambda^(-1/shape)
> perc.obs <- 0.1
> time.cens <- qweibull(perc.obs, mean(shape), mean(scale))
```

```
> dsim <- simCNVdataWeibull(n, mu.surrog, sd.surrog, w, lambda,
+     shape, time.cens)
> head(dsim)

         resp cens cnv     surrog
739  1.482852    0   1  0.1436988
1282 1.482852    0   2  0.8899417
1339 1.482852    0   2  1.6149953
872  1.482852    0   1 -0.2586166
3718 1.482852    0   2  1.2688898
123  1.482852    0   1 -0.9089759
```

The result is a data frame with 4 variables (one additional variable, compared to the Poisson example, that corresponds to censoring indicator), and, as before, as many rows as individuals:

- `resp`: time to disease (weibull distributed) or censoring (end of follow-up),
- `cens`: censoring indicator (0: without disease at the end of follow-up period, 1: with disease within the follow-up period),
- `cnv`: the real copy number status, which, in practice, will be unknown and not considered in testing the association,
- `surrog`: the CNV intensity signal.

As before, the CNV signal is fitted under a normal mixture distribution with function `cnv` and specifying the "mclust" method:

```
> CNV <- cnv(dsim$surrog, mix = "mclust")
> CNV

Inferred copy number variant by a quantitative signal
   Method: function Mclust {package: mclust}

-. Number of individuals: 5000
-. Copies 1, 2, 3
-. Estimated means: 0.0081, 0.9805, 1.9833
-. Estimated variances: 0.1663, 0.1663, 0.1663
-. Estimated proportions: 0.2439, 0.4947, 0.2615



-. Note: number of classes has been selected using the best BIC
```

As for the Poisson example, 1, 2 and 3 copy number have been assigned. So, we need to change the copy number status to 0, 1 and 2 copies, and we proceed as before:

```
> attr(CNV, "num.copies") <- 0:2
> CNV

Inferred copy number variant by a quantitative signal
   Method: function Mclust {package: mclust}

-. Number of individuals: 5000
```

```
-. Copies 0, 1, 2
-. Estimated means: 0.0081, 0.9805, 1.9833
-. Estimated variances: 0.1663, 0.1663, 0.1663
-. Estimated proportions: 0.2439, 0.4947, 0.2615


-. Note: number of classes has been selected using the best BIC
```

Then, an association model with CNV and the phenotype assuming an additive effect is performed as usual, this time specifying `family="weibull"`, and introducing the censored status using function `Surv` in the left hand side of the formula argument: `CNVassoc` function:

```
> fit <- CNVassoc(Surv(resp, cens) ~ CNV, data = dsim, family = "weibull",
+     model = "add")
> coef(summary(fit))
```

```
            HR lower.lim upper.lim         SE     stat       pvalue
trend 1.385556  1.205619  1.592348 0.07097498 4.594595 4.335896e-06
```

Again, the same generic functions are applicable as for normal, binary traits and poisson distributed phenotype. Note that, now, `summary` prints ”HR” instead of ”OR” (binary) or ”RR” (poisson).

References

[1] C. Barnes, V. Plagnol, T. Fitzgerald, R. Redon, J. Marchini, D. Clayton, and M. E. Hurles. A robust statistical method for case-control association testing with copy number variation. *Nature Genetics*, 40(10):1245–52, 2008.

[2] Y. Benjamini and Y. Hochberg. Controlling the false discovery rate: A practical and powerful approach to multiple testing. *J. Roy. Statist. Soc. Ser. B*, 57:289–300, 1995.

[3] J. R. Gonzalez, I. Subirana, G. Escaramis, S. Peraza, A. Caceres, X. Estivill, and L. Armengol. Accounting for uncertainty when assessing association between copy number and disease: a latent class model. *BMC Bioinformatics*, 10:172, 2009.

[4] J. M. Korn, F. G. Kuruvilla, S. A. McCarroll, A. Wysoker, J. Nemesh, S. Cawley, E. Hubbell, J. Veitch, P. J. Collins, K. Darvishi, C. Lee, M. M. Nizzari, S. B. Gabriel, S. Purcell, M. J. Daly, and D. Altshuler. Integrated genotype calling and association analysis of snps, common copy number polymorphisms and rare cnvs. *Nat Genet.*, 40(10):1253–60, 2008.

[5] Myocardial Infarction Genetics Consortium. Genome-wide association of early-onset myocardial infarction with single nucleotide polymorphisms and copy number variants. *Nat Genet*, 41(3):334–341, 2009.

[6] R. M. Neve, K. Chin, J. Fridlyand, J. Yeh, F. L. Baehner, T. Fevr, L. Clark, N Bayani, J-P. Coppe, F. Tong, T. Speed, P. T. Spellman, S. DeVries, A. Lapuk, N. J. Wang, W-L. Kuo, J. L. Stilwell, D. Pinkel, D. G. Albertson, F. M. Waldman, F. McCormick, R. B. Dickson, M. D. Johnson, M. Lippman, S. Ethier, A. Gazdar, and J. W. Gray. A collection of breast cancer cell lines for the study of functionally distinct cancer subtypes. *Cancer Cell*, 10:515 – 527, 2006.

[7] F. Picard, S. Robin, E. Lebarbier, and J. J. Daudin. A segmentation/clustering model for the analysis of array CGH data. *Biometrics*, 63(3):758–766, 2007.

[8] V. Plagnol and C. Barnes. Cnvtools vignette. November 28, 2009.

[9] M. A. van de Wiel, K. I. Kim, S. J. Vosse, W. N. van Wieringen, S. M. Wilting, and B. Ylstra. CGHcall: calling aberrations for array CGH tumor profiles. *Bioinformatics*, 23(7):892–894, 2007.

[10] M. A. van de Wiel and W. N. van Wieringen. CGHregions: dimension reduction for array CGH data with minimal information loss. *Cancer Informatics*, 2:55–63, 2007.
